# Identification of region-specific astrocyte subtypes at single cell resolution

**DOI:** 10.1038/s41467-019-14198-8

**Published:** 2020-03-05

**Authors:** Mykhailo Y. Batiuk, Araks Martirosyan, Jérôme Wahis, Filip de Vin, Catherine Marneffe, Carola Kusserow, Jordan Koeppen, João Filipe Viana, João Filipe Oliveira, Thierry Voet, Chris P. Ponting, T. Grant Belgard, Matthew G. Holt

**Affiliations:** 1 Laboratory of Glia Biology, VIB-KU Leuven Center for Brain and Disease Research, Leuven, Belgium; 2 Laboratory of Glia Biology, KU Leuven Department of Neuroscience, Leuven, Belgium; 3 Life and Health Sciences Research Institute (ICVS), University of Minho, Braga, Portugal; 4 ICVS/3Bs - PT Government Associate Laboratory, Braga, Portugal; 5 IPCA-EST-2Ai, Polytechnic Institute of Cávado and Ave, Applied Artificial Intelligence Laboratory, IPCA Campus, Barcelos, Portugal; 6 Sanger Institute–EBI Single-Cell Genomics Centre, Wellcome Trust Sanger Institute, Hinxton, UK; 7 KU Leuven Department of Human Genetics, Leuven, Belgium; 8 MRC Functional Genomics Unit, Department of Physiology, Anatomy and Genetics, University of Oxford, Oxford, UK; 9 MRC Human Genetics Unit, Institute of Genetics and Molecular Medicine, University of Edinburgh, Edinburgh, UK; 10 Leuven Brain Institute, KU Leuven, Leuven, Belgium; 11 Present Address: The Bioinformatics CRO, Niceville, Florida 32578 USA

**Keywords:** Fluorescence in situ hybridization, RNA sequencing, Astrocyte, Molecular neuroscience

## Abstract

Astrocytes, a major cell type found throughout the central nervous system, have general roles in the modulation of synapse formation and synaptic transmission, blood–brain barrier formation, and regulation of blood flow, as well as metabolic support of other brain resident cells. Crucially, emerging evidence shows specific adaptations and astrocyte-encoded functions in regions, such as the spinal cord and cerebellum. To investigate the true extent of astrocyte molecular diversity across forebrain regions, we used single-cell RNA sequencing. Our analysis identifies five transcriptomically distinct astrocyte subtypes in adult mouse cortex and hippocampus. Validation of our data in situ reveals distinct spatial positioning of defined subtypes, reflecting the distribution of morphologically and physiologically distinct astrocyte populations. Our findings are evidence for specialized astrocyte subtypes between and within brain regions. The data are available through an online database (https://holt-sc.glialab.org/), providing a resource on which to base explorations of local astrocyte diversity and function in the brain.

## Abstract (web summary)

Astrocytes are a major cell type in the central nervous system. Using single cell transcriptome sequencing, the authors identify multiple astrocyte subtypes in the adult mouse CNS, which map to distinct spatial locations and show correlations to cell morphology and physiology.

## Introduction

Astrocytes are ubiquitous in the central nervous system (CNS). They possess thousands of individual processes, which extend out into the neuropil, interacting with neurons, other glia, and blood vessels. Paralleling the wide diversity of their interactions, astrocytes have been reported to play key roles in supporting CNS structure, metabolism, blood–brain barrier formation, and control of vascular blood flow, axon guidance, synapse formation, and modulation of synaptic transmission^1^.

This degree of functional diversity begs the question of whether astrocytes are a homogeneous group of cells or exist in distinct subtypes with specialized functions. Extensive morphological heterogeneity of astrocyte populations was described over 100 years ago in seminal work by Ramón y Cajal^2^. Since then, our understanding of the molecular and cellular heterogeneity of astrocytes has remained largely unaltered: astrocyte classification has largely been restricted to two morphological groupings, fibrous and protoplasmic astrocytes, found in the white and gray matter of the brain, respectively. The question of whether specialized astrocyte subtypes exist remains poorly resolved, largely due to the lack of experimental tools allowing detailed astrocyte characterization^3^. This is in contrast to studies on neurons, for which numerous experimental tools exist and evidence for substantial diversity within brain regions has accumulated^4,5^.

However, the issue of astrocyte diversity is now being addressed and a number of studies are reporting heterogeneity of form and function, both between and within brain regions (see reviews by Khakh and Sofroniew^6^, Ben Haim and Rowitch^7^, Khakh and Deneen^3^ and references therein). The expression of fluorescent reporter tags in astrocytes has allowed the isolation of cells from specific brain regions for RNA profiling^8,9^ and proteomic studies^10^. Fusion of reporter tags to ribosomal subunits (TRAP technology) has permitted the isolation of actively translated mRNAs from astrocytes^10–12^. Together, these studies revealed that gene expression varies between brain regions, and that there is often a subtle gradient of gene expression within individual brain areas. In addition, astrocytes in different brain regions also have distinct morphological and functional features, such as degree of synapse association^9,10^, intrinsic membrane properties and Ca^2+^ signaling^10^, and ability to promote neuronal maturation^12^. Refined labeling strategies using promoter fragments^13^, or intersectional approaches^14,15^ to isolate subpopulations of cells have revealed intra-regional heterogeneity in the cortex^13–15^, as well as the brainstem, thalamus, olfactory bulb, cerebellum and spinal cord^14^, which again correlates to morphology^15^, cell intrinsic physiology^15^ and function^13,14^.

Astrocytes are also involved in disease, as evidenced by extensive cell culture and mouse model studies^6^, with disruption of astrocyte functions, such as synapse formation, leading to neuronal network dysfunction^13,14^. Astrocyte heterogeneity may underpin the differential transcriptomic responses seen to bacterial infection (lipopolysac charide, LPS) treatment and stroke (middle cerebral artery occlusion, MCAO)^16^ in mouse astrocytes, as well as in response to demyelination in the experimental allergic encephalomyelitis (EAE) model^17^, suggesting that successful treatment may have to take account of cell type and brain region, insult, and insult severity. One possible reason for the regional difference in tumor susceptibility in the CNS is the location-dependent ability of astrocytes to proliferate, due to differential expression of tumor suppressor genes^18^. Given that analysis of gene co-variation patterns from human tissue samples indicates regional specific astrocyte subtypes^19^, it is possible that heterogeneity plays a role in human conditions, such as Norrie disease^13^.

Single-cell analysis approaches are revolutionizing our concepts of cell identity and heterogeneity. Widely used in the CNS, they have revealed a high diversity of neuronal subtypes across brain regions^4,5^. In comparison, astrocyte diversity is reported to be low. Whether this reflects the true nature of astrocytes, or is due to technical issues such as low RNA content^20^, remains an open question. Consequently, to address the diversity of astrocyte types in the adult mouse brain, we systematically optimized the Smart-seq2 protocol^21^, including changes to cell isolation steps and library preparation. We used this pipeline to obtain full-length coverage of cDNAs prepared from thousands of individual astrocytes from adult mouse cortex and hippocampus, areas selected for their well-studied anatomy, physiology, and broad disease relevance. Cluster-based analysis revealed five molecularly distinct astrocyte subtypes, which were confirmed in situ in the mouse brain. By mapping the spatial position of each subtype, we found a more refined organization of CNS tissue than previously anticipated, with unique molecular astrocyte subtypes occupying distinct positions, suggestive of specific intra-regional functions. Furthermore, this spatial patterning correlated to the positions of astrocytes with unique morphologies and Ca^2+^ signaling. Our data provide a valuable resource for future hypothesis-driven experiments, aimed at dissecting out the contributions of astrocyte subtypes to CNS function—and are freely available in a standalone database accessible at https://holt-sc.glialab.org/. Such insights might be important to our future understanding of regional susceptibility to diseases, such as Alzheimer’s and Parkinson’s, in which astrocytes are increasingly implicated^22^.

## Results

### Single-cell mRNA sequencing reveals astrocyte heterogeneity

**Fig. 1:**
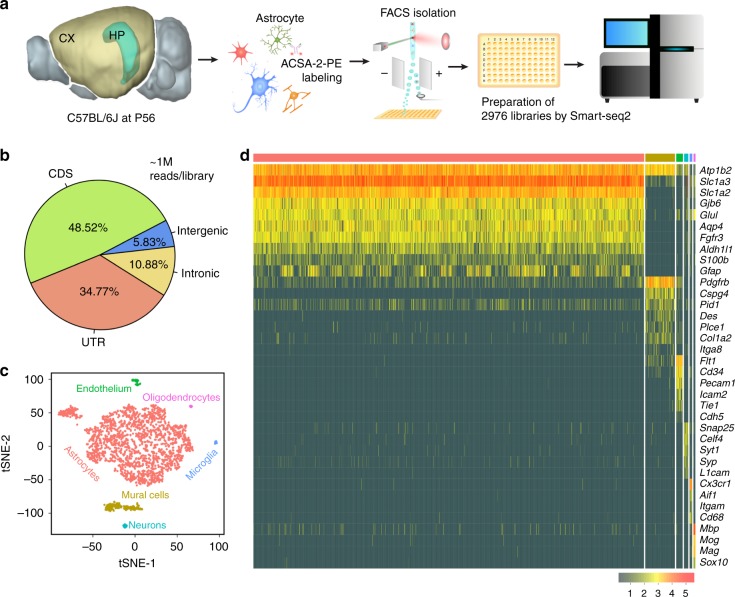
Single-cell sequencing strategy and cell-type identification. **a** Whole brains were obtained from C57BL/6J mice at postnatal (P) day 56. Cortical (CX) and hippocampal (HP) astrocytes were prepared separately, using enzymatic digestion followed by mechanical trituration. Two separate batches of astrocytes for each region were prepared. Cortical cell suspensions were prepared from two littermate animals in parallel using separate tubes. Hippocampal cell suspensions were also prepared in parallel using separate tubes; in this case, two different sets of four littermate animals were used. Astrocytes were then specifically labeled with the ASCA-2-PE antibody and single cells were deposited in individual wells of a PCR plate using FACS. Single-cell library preparation was performed using a modified Smart-seq2 protocol. In total, 2976 libraries were prepared and sequenced using a NextSeq 500 system (Illumina). **b** Each library was sequenced to optimal coverage (on average 1 M reads per library). In total, 2015 high-quality libraries were retained for further analysis. In these libraries, a high fraction of reads mapped to exons (CDS, coding sequence; UTR, untranslated region). Conversely, a low fraction of reads mapped to intronic and intergenic regions. **c** Visualization of the major higher-order cell types (2015 cells) identified by Seurat using tSNE plots. Each dot represents a single cell. Cells with similar molecular profiles group together; cell types were assigned according to the expression of specific marker genes (and are labeled in different colors). **d** Gene expression heatmap for higher-order cell types (columns) grouped according to the Seurat classification shown in Fig. 1c. Color-coding from Fig. 1c is retained. Gray, no expression; yellow, low expression; red, high expression, In-normalized gene expression data is shown.

To obtain an unbiased and comprehensive comparison of astrocytes in mouse cortex and hippocampus, we performed single-cell RNA sequencing. Although dissociating adult mammalian brain tissue into healthy and representative cell suspensions is challenging, astrocytes show transcriptional changes during development (such as in glutamatergic signaling)^23^, which can obscure gene expression differences underlying the functional specialization of cell types^5^. To avoid these issues, we recovered the cortex and hippocampus from C57BL/6J mice aged to postnatal (P) day 56, which should avoid introducing bias from transcriptional programs associated with development^5^, while maintaining compatibility with external gene expression databases, such as the Allen Brain Atlas^24^. A single-cell suspension for each brain region was obtained using a papain-based protocol, which was previously shown to give good tissue dissociation, with high levels of cell viability^25^. As adult brain is heavily myelinated, a Percoll gradient was used to reduce contamination, while ensuring efficient cell recovery (Supplementary Fig. 1 and Supplementary Note 1). Astrocytes were then labeled with the ACSA-2 antibody (conjugated to the fluorophore phycoerythrin (PE)). This antibody specifically recognizes the plasma membrane marker ATP1B2, which is detected at both the mRNA^20^ and protein^25^ levels in astrocytes through the adult mouse cortex and hippocampus (Supplementary Fig. 2). As mature oligodendrocytes express low levels of ATP1B2, staining with an anti-O1 antibody conjugated to eFluor660 was also performed (Supplementary Fig. 3). Viable astrocytes (as determined with 7-amino-actinomycin D (7-AAD) staining) were isolated by fluorescence-activated cell sorting (FACS), with one cell deposited per single well of a PCR plate (Supplementary Fig. 4). Preparation of sequencing libraries was done using the Smart-seq2 protocol^21^, in which the concentration of template switching oligonucleotide (TSO), number of PCR preamplification cycles, and the DNA clean-up step were optimized for use with cells with low RNA content^21^ (Fig. 1a, Methods, Supplementary Figs. 5 and 6, and Supplementary Note 1). In all, 2976 individual libraries were sequenced to optimal coverage (~1 million reads per cell) (Fig. 1b). Low-quality libraries were removed based on FastQC and additional quality metrics (see Methods and Supplementary Fig. 7), leaving 2015 high-quality libraries. On average, each of these contained 83% exonic reads, mapping to 2148 genes, with only 11% intronic and 6% intergenic reads (Fig. 1b).

**Fig. 2:**
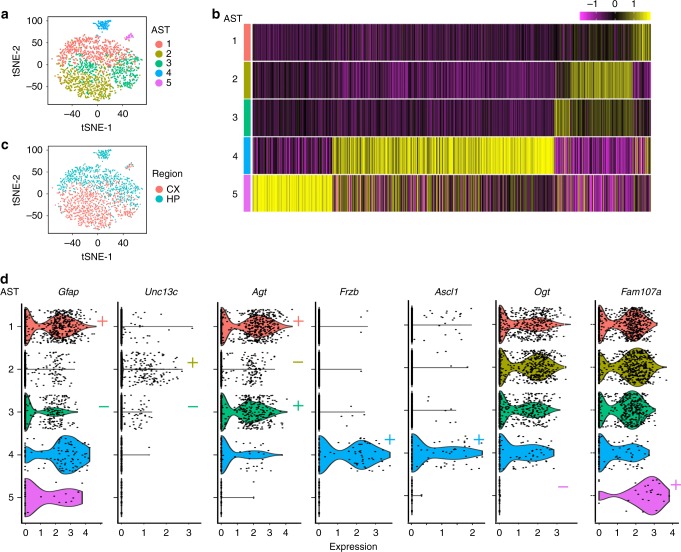
Identification of astrocyte subtypes in adult mouse cortex and hippocampus. Single-cell data were used to identify distinct astrocyte subtypes (AST). **a** A total of 1811 astrocytes were identified from higher-order clustering. This data was extracted and reclustered using Seurat and five distinct astrocyte subtypes were identified. Clusters are presented in tSNE plots, with each AST color-coded. **b** Hierarchically clustered average gene expression heatmap for genes overexpressed across subtypes. Rows correspond to cells, columns to genes. Magenta, low gene expression; yellow, high expression. Scaled ln-normalized data are shown. **c** Astrocytes derived from the cortex (CX) or hippocampus (HP) segregate based on gene expression. **d** Expression of subtype-specific marker genes selected for in situ hybridization experiments. Markers are classed as absent/low “−” or highly expressed “+,” based on ln-normalized expression data. See also Supplementary Data.

Clustering analysis was performed using Seurat^26^. Analysis of these libraries revealed a residual fraction of contaminating higher-order CNS cell types (Fig. 1c, d and Supplementary Table 1). Cell-type identification was based on the expression of known marker genes (Fig. 1c). Following removal of contaminating cell types, 1811 astrocytes remained. These cells were then reclustered, based on the 886 highly variable genes expressed across astrocytes (see Methods for more details). This led to the identification of five distinct Astrocyte SubTypes (AST1–5), each distinguished by a gene expression fingerprint (Fig. 2a, b). T-distributed stochastic neighbor embedding (tSNE) plots showed three major “clouds” of cells. AST4 and AST5 formed distinct clusters, suggesting distinct molecular fingerprints. In contrast, ASTs 1–3 were grouped together, suggesting much more subtle differences in gene expression between these subtypes (Fig. 2a and Supplementary Fig. 8). Astrocyte subtypes constituted different proportions of the total cell population analyzed using our optimized Smart-seq2 protocol, ranging from 1.4% (AST5) to 36.5% (AST1) (Supplementary Table 2).

As expected, there was clear separation of subtypes between the cortex and hippocampus^11,12^ (Fig. 2c and Supplementary Table 2), with AST1 and AST4 being predominantly hippocampal, AST2 being mainly cortical, and AST3 and AST5 being distributed uniformly between brain regions. As control experiments effectively excluded clustering by batch effects during sample processing (Supplementary Data 1 and https://holt-sc.glialab.org/sc/), these data confirmed that our protocol could identify both inter- and intra-regional heterogeneity of astrocytes in adult mouse brain.

### Unique molecular signatures define astrocyte subtypes

**Fig. 3:**
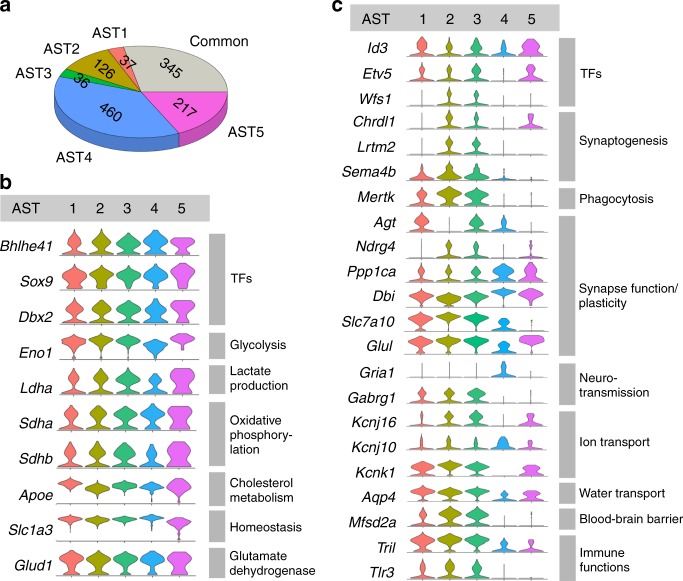
Identification of common and differentially expressed genes in astrocytes. **a** Chart showing the number of genes expressed in at least 60% of sampled astrocytes (common) and the number of genes specifically enriched in each subtype. **b** Examples of genes common across astrocyte subtypes, classified by biological function. TFs, transcription factors. **c** Examples of genes highly enriched in specific astrocyte subtypes, classified by biological function. Note, some genes, e.g. *Gabrg1* (γ-aminobutyric acid type A receptor γ1 subunit), could be classified as either an ion channel or as involved in synaptic function/plasticity. Here, classification was based on the principal identified function—ion channel activity.

Analysis of sequencing data showed a number of genes common across astrocytes (expressed in >60% of cells). However, the astrocyte subtypes we identified also showed enrichment of specific genes (Figs. 2d and 3a). To gain insight into the possible roles played by differential gene expression, we analyzed transcript lists using gene enrichment and functional annotation (DAVID)-based approaches^27^, and manual curation using the UniProt database^28^.

Genes commonly expressed across astrocytes (Supplementary Data 2) include transcription factors known to play a role in neural patterning (*Dbx2*) and astrocyte specification (*Sox9*). Perhaps unsurprisingly, the majority of remaining common genes were associated with energy production through glycolysis and oxidative phosphorylation (*Eno1*, *Sdha*, *Sdhb*). Energy supply as a common function was further indicated by genes associated with lactate production (*Ldha*). Cholesterol synthesis and trafficking (*Apoe*), glutamate uptake (*Slc1a3*), and glutamate metabolism (*Glud1*) have also been described as common astrocyte processes^1^ (Fig. 3b).

However, >70% of enriched genes were specific to a subtype (Supplementary Table 3 and Supplementary Data 2–4). Furthermore, only one gene among the top ten expressed by each subtype was shared (Supplementary Table 4). Among differentially expressed genes, transcription factors (*Id3*, *Etv5*, *Wfs1*) were prominent, consistent with diverse transcriptional networks maintaining unique cell identities. Consistent with this, differential expression of specific genes was found across all major astrocyte functions (chosen based on a number of recent reviews)^6,7,29–32^. These functions include synaptogenesis (*Chrdl1*, *Lrtm2*, and *Sema4b*), phagocytosis (synapse removal) (*Mertk*), synapse function/plasticity (*Agt*, *Ndrg4*, *Ppp1ca*, *Dbi*, *Slc7a10*, and *Glul*), neurotransmission (*Gria1* and *Gabrg1*), ion transport (*Kcnj16*, *Kcnj10*, and *Kcnk1*), water transport (*Aqp4*), formation and/or maintenance of the blood–brain barrier (*Mfsd2a*), and immune function (*Tril* and *Tlr3*) (Fig. 3c).

Hence, our data further confirm the molecular heterogeneity of astrocytes. This heterogeneity encompasses well-recognized astrocyte functions and exists both between and within brain regions.

### In situ mapping of astrocyte subtype location

Based on closer examination of RNA-seq gene lists, a set of markers was identified to specifically label astrocytes (Fig. 2d), based on overall levels of gene expression (absent/low to high relative expression). These genes encoded proteins involved in cytoskeletal function (*Gfap*), membrane fusion (*Unc13c*), regulation of cerebral blood flow and synaptic transmission (*Agt*), Wnt signaling (*Frzb*), cell fate specification (*Ascl1*), post-translational protein modification (*Ogt*), and actin binding (*Fam107a*), respectively. To specifically map back astrocyte subtypes in brain tissue, we performed multiplexed fluorescence in situ hybridization (ISH) experiments (RNAscope) on coronal sections of adult mouse brain, using probes specific for these markers (see Methods and Supplementary Table 5).

### A unique subtype linked to neurogenesis: AST4

**Fig. 4:**
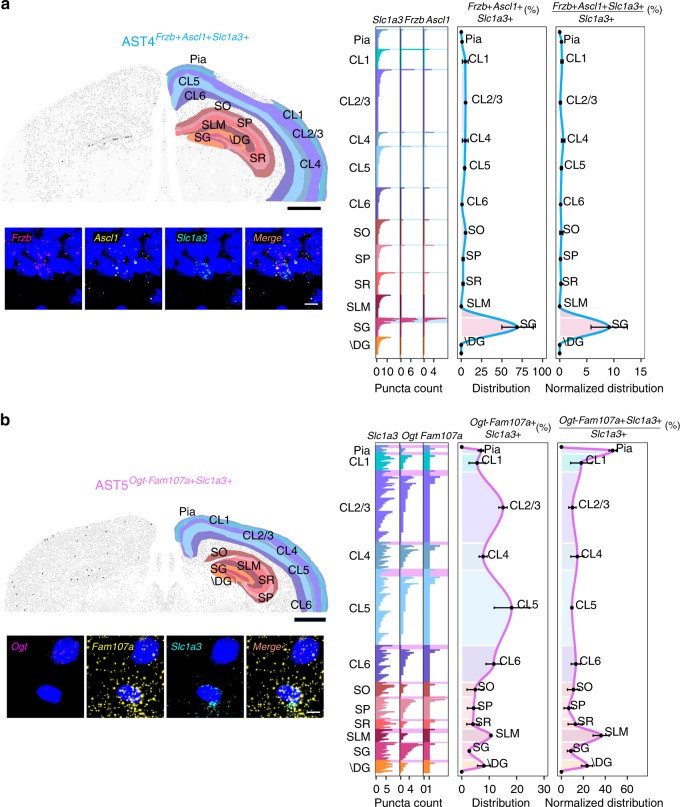
Differential patterning of AST4 and AST5 in adult mouse brain. Multiplexed fluorescence in situ hybridization was used to map locations of AST4 and AST5. **a** AST4 was identified by high expression levels of *Frzb, Ascl1*, and *Slc1a3*. **b** AST5 was identified by absence/low expression of *Ogt* and high expression of both *Fam107a* and *Slc1a3*. Mapping was performed on three sections obtained from three independent animals aged between P56–P60. Representative images are shown. Top left: low-magnification image of a coronal section. Black dots show the distribution of the astrocyte subtype through one brain hemisphere. Brain regions are defined manually based on definitions from the Allen Brain Atlas. High-magnification images (below) show the localization of markers to specific cells defined on the basis of nuclear (DAPI, blue) staining. Right: bar plots (showing from left to right) fluorescence counts per RNA marker per cell (shown for all cells across all sections analyzed), the distribution of the subtype between brain regions and the distribution of the subtype normalized to the total number of astrocytes per brain region (all *Slc1a3* + cells). Astrocytes belonging to the subtype of interest are highlighted by a shaded box (color-coded according to the scheme used in Fig. 2a). Astrocyte numbers across layers are given as average per section analyzed. Error bars are equivalent across the figure and represent SEM. Scale bars, low magnification 1000 µm; high magnification, 10 µm. “+” high gene expression, “−” low or absent gene expression. SO Stratum oriens, SP Stratum pyramidale, SR Stratum radiatum, SG Subgranular zone, \DG Dentate gyrus without SG, SLM Stratum lacunosum-moleculare.

Examination of genes enriched in AST4 revealed a disproportionate number of them to be involved in mitosis and cell cycle control (*Cdk4* and *Sirt2*), transcriptional regulation (*Ascl1* and *Emx1*), and neurogenesis and neuronal differentiation (*Dab1*) (Supplementary Tables 6 and 7). Our initial tissue dissection also recovered the dentate gyrus of the hippocampus. Based on the high expression levels of *Frzb*, *Ascl1*, and *Slc1a3* in our sequencing data, the fact that *Ascl1* is known to be expressed in neural stem cells and amplifying progenitors, and the known staining patterns of these genes in the Allen Brain Atlas (Supplementary Fig. 9), we hypothesized that AST4 represents a population of hippocampal neural stem or progenitor cells^33,34^. Coronal sections of adult mouse brain were stained with probes against *Frzb* and *Ascl1* as subtype-specific markers and *Slc1a3* as a general marker of stem cells and astrocytes^34^ (Fig. 4a and Supplementary Figs. 10 and 11). The anatomical distribution of cells expressing all three marker genes is shown in the low-magnification section, using black dots to mark cells of interest. To allow a detailed description of astrocyte localization and quantification, images were manually segmented, based on definitions from the Allen Brain Atlas (Mouse Reference Atlas, Coronal). Higher magnification images confirming colocalization to individual cell nuclei are also shown, with quantification of individual fluorescent puncta per cell used as a proxy for mRNA expression levels (left hand bar plot Fig. 4a and Methods). The distribution of AST4 throughout the brain was quantified in two separate ways. First, distribution through the brain was plotted, based on the number of AST4 astrocytes detected in a given region (middle plot, Fig. 4a). Second, the proportion of AST4 astrocytes relative to the total number of all astrocytes in each brain region was determined (right hand plot, Fig. 4a). As predicted, AST4 localizes predominantly to the subgranular zone in the hippocampus and forms the majority of *Slc1a3*-positive cells detected in this region. This result verifies the power of our sequencing-based approach to resolve individual astrocyte subtypes, as well as our ISH-based mapping.

### A possible intermediate progenitor: AST5

AST5 was the rarest subtype we found by Smart-seq2 sequencing (Fig. 2a and Supplementary Table 2). AST5 showed considerable overlap with AST4 (Supplementary Table 3), being enriched in genes involved in mitosis and cell cycle control (*Sirt2*, *Sept2*, *Emp2*, etc). However, there were also considerable differences, with AST5 being enriched for genes involved in classical astrocyte functions, such as glucose metabolism and energy production (Supplementary Tables 6 and 8), suggesting that AST5 represents an intermediate transition state between progenitors and mature astrocytes. The distribution of AST5, based on relatively high expression of *Fam107a* and low expression/absence of O*gt* (Fig. 4b and Supplementary Figs. 10 and 11), was difficult to obtain accurately given the large variability between samples. However, based on absolute cell numbers, a trend exists towards enrichment in cortical layers 2/3 and 5. As a proportion of the *Slc1a3*-positive cell population, AST5 appeared dominant in the subpial region, as well as the stratum lacunosum-moleculare and dentate gyrus of hippocampus.

### Mature astrocytes: ASTs 1, 2, and 3

In contrast, AST1, AST2, and AST3 showed gene-enrichment profiles more consistent with mature astrocyte function.

**Fig. 5:**
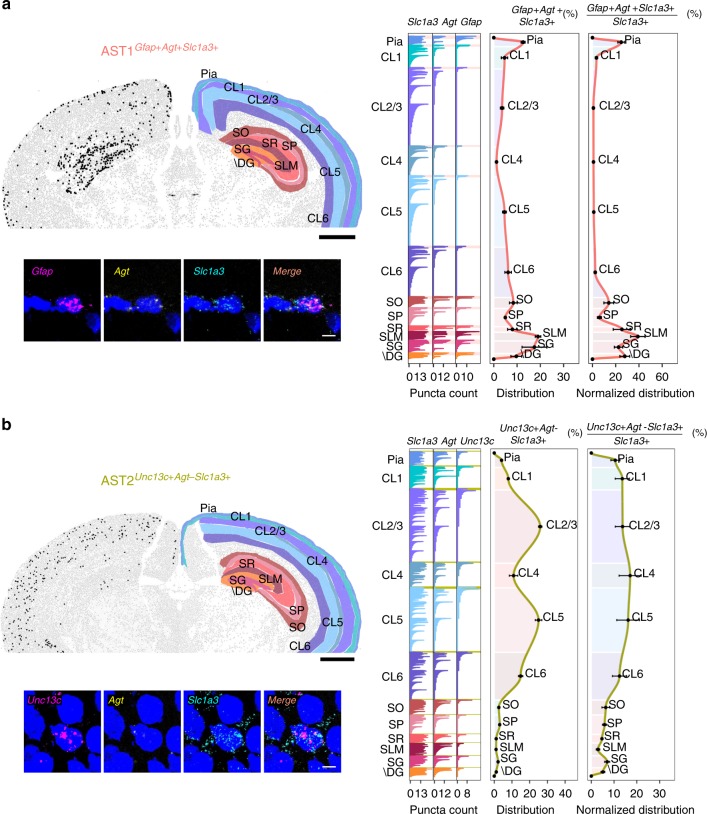
Differential patterning of AST1 and AST2 in adult mouse brain. Multiplexed fluorescence in situ hybridization was used to map locations of AST1 and AST2. **a** AST1 was identified by high expression levels of *Gfap*, *Agt*, and *Slc1a3*. **b** AST2 was identified by low expression/absence of *Agt* and high expression of both *Unc13c* and *Slc1a3*. Mapping was performed on three sections obtained from three independent animals aged between P56 and P60. Representative images are shown. Top left: low-magnification image of a coronal section. Black dots show the distribution of the astrocyte subtype through one brain hemisphere. Brain regions are defined manually based on definitions from the Allen Brain Atlas. High-magnification images (below) show the localization of markers to specific cells defined on the basis of nuclear (DAPI, blue) staining. Right: bar plots (showing from left to right) fluorescence counts per RNA marker per cell (shown for all cells across all sections analyzed), the distribution of the subtype between brain regions, and the distribution of the subtype normalized to the total number of astrocytes per brain region (all *Slc1a3* + cells). Astrocytes belonging to the subtype of interest are highlighted by a shaded box (color-coded according to the scheme used in Fig. 2a). Astrocyte numbers across layers are given as average per section analyzed. Error bars are equivalent across the figure and represent SEM. Scale bars, low magnification 1000 µm; high magnification, 10 µm. “+” high gene expression, “−” low or absent gene expression. SO Stratum oriens, SP Stratum pyramidale, SR Stratum radiatum, SG Subgranular zone, \DG Dentate gyrus without SG, SLM Stratum lacunosum-moleculare.

AST1 is defined by high expression of *Gfap* and *Agt*, and was found at high levels in the subpial layer and hippocampus—both in terms of absolute distribution and normalized to total astrocyte number (Fig. 5a and Supplementary Figs. 10, 12, and 13). This is entirely consistent with previous reports of *Gfap* staining in the rodent brain^30^ and the unique characteristics of marginal astrocytes^9,35^. Gene enrichment and functional annotation analysis revealed only a handful of subtype overexpressed genes and related pathways (Supplementary Tables 6 and 9). With reference to common astrocyte functions, however, synaptogenesis (*Nrxn1*, *Prex2*, and *Plekhb1*), synaptic plasticity (*Agt*), and glutamatergic neurotransmission (*Arl6ip1*) were clearly distinct from other subtypes (Supplementary Table 9).

**Fig. 6:**
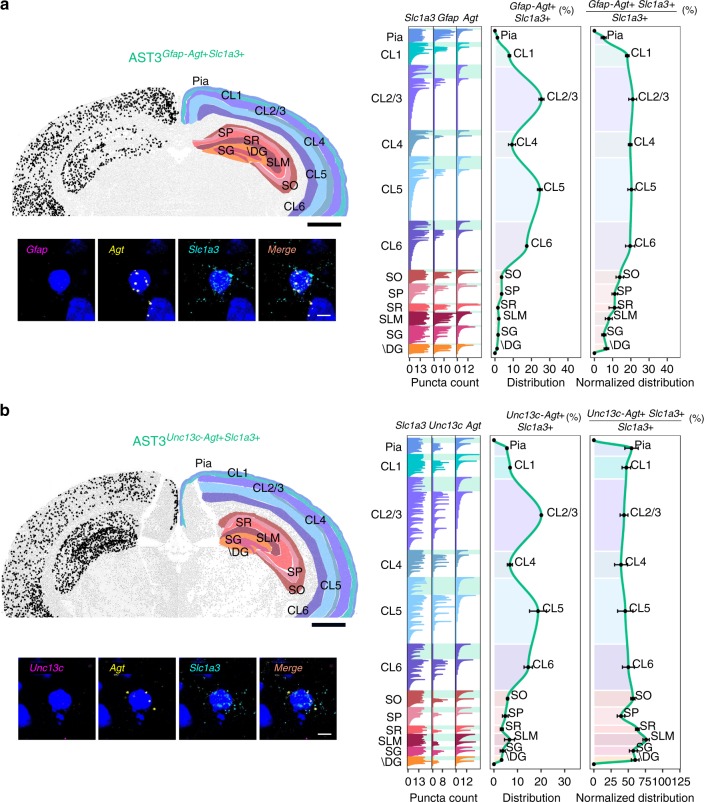
Differential patterning of AST3 in adult mouse brain. Multiplexed fluorescence in situ hybridization was used to map the location of AST3. Due to technical limitations, AST3 was mapped using a split marker approach. Sections were assessed for (**a**) low expression/absence of *Gfap* with expression of *Agt* and *Slc1a3* (to differentiate AST3 from AST1) and (**b**) low expression/absence of *Unc13c* with expression of *Agt* and *Slc1a3* (to discriminate between AST3 and AST2). Mapping was on three sections from three independent animals aged P56–P60. Representative images are shown. Top left: low-magnification image of a coronal section. Black dots show astrocyte subtype distribution through one hemisphere. Regions are defined manually based on the Allen Brain Atlas. High-magnification images (below) show the localization of markers to specific cells based on nuclear (DAPI, blue) staining. Right: bar plots (showing left to right) fluorescence counts per marker per cell (for all cells across all sections analyzed), the distribution of the subtype between brain regions and the distribution of the subtype normalized to the number of astrocytes per brain region (all *Slc1a3* + cells). Astrocytes belonging to the subtype of interest are highlighted by a shaded box (color-coded according to the scheme used in Fig. 2a). Astrocyte numbers across layers are given as average per section analyzed. Error bars are equivalent across the figure and represent SEM. Scale bars, low magnification 1000 µm; high magnification, 10 µm. “+” high gene expression, “−” low or absent gene expression. SO Stratum oriens, SP Stratum pyramidale, SR Stratum radiatum, SG Subgranular zone, \DG Dentate gyrus without SG, SLM Stratum lacunosum-moleculare.

**Fig. 7:**
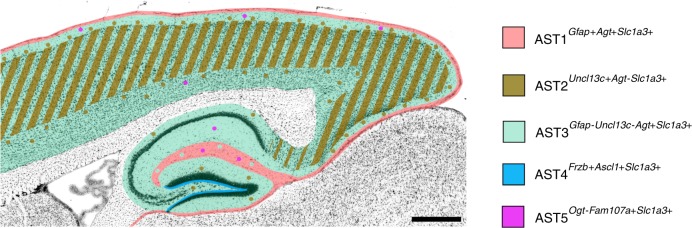
Schematic summary of astrocyte subtype positions in adult mouse brain. Indicated positions are based on in situ hybridization data (Figs. 4–6) and are marked on a representative sagittal section of adult mouse brain (adapted from the Allen Mouse Brain Atlas). Subtypes are color-coded (as in Fig. 2a). Scale bar, 500 µm.

Based on ISH, both AST2 and AST3 showed highly reproducible and specific distribution patterns across cortical layers and the hippocampus. AST2 was localized by higher than average expression of the marker gene *Unc13c* and low or absent expression of *Agt*. It was found in the highest absolute numbers in cortical layers 2/3 and 5, with lower amounts in layers 1, 4, and 6, and negligible amounts in the hippocampus (Fig. 5b and Supplementary Figs. 10 and 14). However, normalized to total astrocyte number per region, AST2 appears uniformly distributed across cortical layers. The distribution of AST3 was predicted by expression of *Agt* and little or no expression of *Unc13c* and *Gfap*. The limited number of spectral channels available to us at the time using RNAscope (three markers including *Slc1a3*) meant we had to adopt a split-staining approach, performing two different sets of staining: the first for *Gfap*, *Agt*, and *Slc1a3* (Fig. 6a) and a second for *Unc13c*, *Agt*, and *Slc1a3* (Fig. 6b). Both showed AST3 distributed throughout the cortex and hippocampus (see also Supplementary Figs. 10, 12-14). Based on the high levels of AST1 localizing to the pial layer and stratum lacunosum-moleculare in the hippocampus (Supplementary Fig. 12), we anticipate it being the dominant subtype in these regions. Considering the heavy *Gfap* staining, and the split staining approach taken for AST3, we expect that the overall levels of AST3 are relatively low in these two regions (Fig. 6a vs. Fig. 6b). It is possible that AST2 also follows a similar distribution pattern in the cortex, and is found in lower amounts in the pial region, as *Gfap* expression is also low in AST2 (Fig. 2). RNAscope stainings across multiple tissue sections (Supplementary Fig. 10) suggest a substantial degree of intermixing between these two cell types in mid-cortical layers, while AST3 appears to be the dominant subtype in layer 6. In this respect, it is interesting that the two subtypes show differential gene-enrichment profiles for processes relating to synaptic function (AST2, glutamatergic transmission, *Slc7a10*, *Gria2*; AST3, GABAergic transmission, *Gabrg1*) and synaptogenesis and process outgrowth (AST2, *Slitrk2*, *Sema4b*; AST3, *Etv5*, and *Spon1*), suggesting differential regulation of functions by these subtypes (Supplementary Table 9). For ease of interpretation, we have summarized the complex spatial relationships between subtypes schematically (Fig. 7).

### Morphological correlates of identified astrocyte subtypes

Distinct transcriptomic profiles and spatial locations for astrocyte subtypes predict morphological and functional specialization^7^. Unfortunately, with the exception of *Gfap*, the markers exploited for subtype identification currently have a limited range of molecular reagents available (green fluorescent protein (GFP)-marker mice, specific Cre-lines, antibodies, etc.) to facilitate further such experiments. However, our ISH-based mapping showed that subtypes are differentially distributed across the cortex and hippocampus. Therefore, we used an alternative strategy to investigate these issues, based on the sampling of large numbers of astrocytes in specific brain regions. Our rationale was that by taking this approach we could correlate the presence of distinct morphologies or functional characteristics to the spatial patterning of our molecular subtypes. Given the limitations of our approach, we decided to focus on regions containing ASTs 1, 2, and 3, as these subtypes most likely represent mature astrocytes.

Previous studies on astrocyte morphology have given variable results, presumably due to the labeling methods used. Hence, we examined recent studies in which either regional or sub-regional differences were measured, using methods that gave high astrocyte coverage in adult C57BL/6 mice^9,36^.

Astrocytes positioned in cortical layers 2–4 (which contain AST2 and AST3) display a smaller territorial volume and arborization than astrocytes in the hippocampal CA3 region (containing AST3), as judged by Golgi staining and immunohistochemistry for the astrocyte marker S100β^36^.

Cortical astrocytes, labeled using genetically encoded marker proteins, show differences in arborization, territorial volume, and cell orientation through layers 1–6^9^. These become more apparent using a hierarchical clustering of five key morphological parameters (including elongation, flatness, and various measures of cell orientation)^9^. Using this approach, the authors identified four prominent morphological subtypes (A–D), which distribute through the various cortical layers in differing proportions^9^, consistent with data from our ISH experiments (Figs. 4–6). Of note, there is a correlation between the morphologies identified and the molecular subtypes we report. Marginal astrocytes (AST1) showed a substantial similarity to Cluster D, given their unique morphology and localization in the pial layer^9,37^. Cluster B was present in cortical layers 1–6, whereas Cluster C was present in cortical layers 2–5, with lower proportions in layers 1 and 6. These distributions show remarkable similarities to those of AST3 and AST2, respectively. However, Cluster A was restricted to layers 5 and 6, suggesting that the correspondence between transcriptome and morphology is incomplete (see below).

### Physiological correlates of identified astrocyte subtypes

**Fig. 8:**
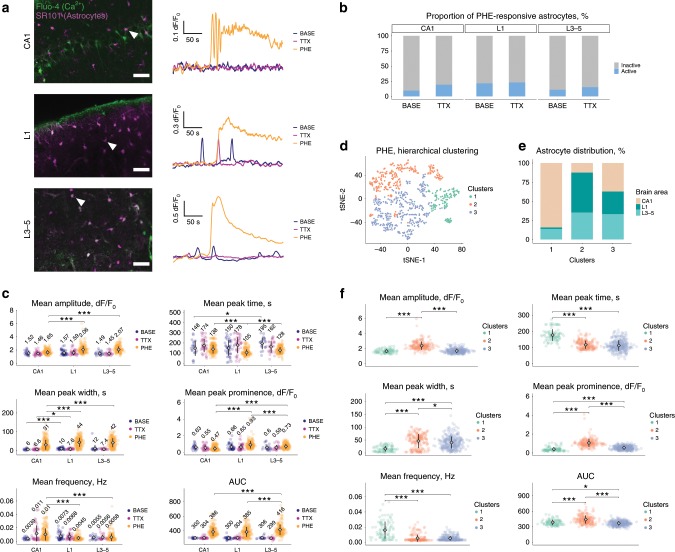
Astrocyte subpopulations display distinct Ca^2+^ transient properties. Calcium transients in SR101-labeled astrocytes were detected using Fluo-4. Measurements were made in acute brain slices containing cortical layer 1 (L1), cortical layers 3–5 (L3–5), and the CA1 region of the hippocampus (CA1). Transients were recorded under sequential conditions of baseline activity (BASE), tetrodotoxin (TTX), and TTX plus phenylephrine (PHE). **a** Representative astrocytes (arrowheads) and the calcium transients recorded from them under each experimental condition. Scale bar, 50 µm. **b** The total population of active astrocytes was defined as cells responding to application of PHE. The fraction of this population displaying Ca^2+^ transients under BASE and TTX conditions is shown in blue. **c** Transient parameters grouped by the brain region recorded. Numerical values are the means for each condition. **d** Hierarchical clustering of Ca^2+^ transient parameters after application of PHE. **e** Proportion of astrocytes from the various brain regions per cluster defined in **d**. **f** Astrocyte peak parameters grouped per cluster. One dot equals one cell in **c**, **d**, and **f**. Plots in **c** and **f** show mean ± SD. Data normality was tested using a Shapiro–Wilk test. Significant differences were verified using a Kruskal–Wallis test with post-hoc Dunn’s test, with *p*-values adjusted with the Benjamini–Hochberg method. *p ≤ 0.05, **p ≤ 0.01, ***p ≤ 0.001. AUC area under the curve. Nine animals were used. In total, 614 cells were analyzed.

Astrocyte activation is commonly associated with a transient rise in intracellular Ca^2+^, which has been linked to functional outputs^6^. Hence, heterogeneity in Ca^2+^ signaling has been proposed as a mechanism by which astrocytes execute diverse functions^6^. We investigated the possibility of differential astrocyte Ca^2+^ signaling using acute coronal tissue slices, in which cells were labeled with the Ca^2+^ indicator Fluo-4, and co-labeled with sulforhodamine 101 (SR101) for astrocyte identification. Images were acquired using a two-photon microscope in cortical layer 1, cortical layers 3–5, and in hippocampal CA1. Cell activity was measured under three consecutive conditions. Baseline (BASE) reflects astrocyte activity when neurons were spontaneously active. Application of tetrodotoxin (TTX) was then used to block neuronal activity, isolating astrocytes from the influence of local neuronal network activity. Finally, phenylephrine (PHE) was applied to TTX-treated slices to directly trigger robust Ca^2+^ responses in astrocytes^38^, independent of neuronal activity (Fig. 8a). Only cells responding to PHE were retained for analysis (Fig. 8b) and profiles showing changes in intracellular Ca^2+^ were plotted as fractional fluorescence changes relative to baseline (dF/F_o_) (Fig. 8a). Although we observed significant differences in activity between astrocytes across regions in baseline and TTX conditions, the most striking differences were seen after application of PHE (Fig. 8c).

To eliminate potential bias, caused by manual assignment of cells to brain regions, we performed unbiased hierarchical clustering of astrocytes, based on the various Ca^2+^ peak parameters recorded following PHE application (Fig. 8d, e). We identified three distinct clusters of astrocytes, which were differentially distributed through cortical layers 1 and 3–5, as well as hippocampal CA1. Cluster 2 mostly comprised cortical astrocytes from layer 1 and layers 3–5. In contrast, hippocampal astrocytes were a minority. Based on comparisons to our ISH data, it is likely that Cluster 2 contains a mixture of AST1 and AST2, which are located in the cortex. Cluster 3 contained roughly equal quantities of astrocytes from across brain regions, consistent with cells having an AST3 identity. Interestingly, Cluster 1 was mostly composed of hippocampal CA1 astrocytes and likely comprised AST3, indicating the potential for further axes of heterogeneity, such as local environment, to also influence cell identity (see below). Significant differences across clusters were seen for the measured signal parameters (Fig. 8f).

Taken together, these data indicate that molecularly defined astrocyte subtypes share overlapping spatial locations with cells possessing distinct morphologies and physiological responses.

## Discussion

Astrocytes have been reported to play many diverse roles in the CNS, such as synapse formation and elimination, maintenance of local homeostasis, and modulation of synaptic transmission^1^. However, whether astrocytes are functionally specialized to perform certain tasks remains unclear. This is despite evidence supporting the formation of local specialized neuronal microcircuits, in areas such as visual cortex^39^ and hippocampus^40^.

To measure diversity both between and within brain regions, we used a single-cell sequencing approach in adult tissues to achieve an unbiased view of astrocyte heterogeneity, free of possible interference from transcriptional programs related to CNS development^5^. Our results distinguish multiple astrocyte subtypes at the transcriptomic level. However, the picture is substantially more complicated than for neurons, with little clear evidence for distinct cellular hierarchies in clustering, most likely due to multiple axes of heterogeneity being present^4,5,20,41^. However, although astrocyte subtypes share common genes associated with core functions, the transcriptomic differences between the subtypes are still sufficiently large to suggest distinct specializations in known astrocyte functions^13,42^. This is reinforced by our ISH results. Differential localization through the hippocampus and cortex supports the concept of local astrocyte diversity, whereas consistency across sections from multiple animals indicates the subtypes identified are genuine and not transient cell states. As we performed analysis on sections cut to maximize the cross-sectional area of the cortex and hippocampus for imaging (Supplementary Fig. 10), we cannot discount variations in spatial distribution along the rostro-caudal axis. However, as the cells recovered for sequencing were obtained from the whole cortex and hippocampus, we are confident the overall subtype distribution will largely reflect this fact.

The observation of unique transcriptomic profiles and spatial patterning suggests several intriguing possibilities, including links between cortical and hippocampal astrocyte populations. Astrocyte generation from distinct progenitors in mouse brain and spinal cord has been proposed, in which broad sections of the CNS are populated by astrocytes derived from progenitors of fixed developmental origin^43,44^. For example, the widespread distribution of AST1 and AST3 across the cortex and hippocampus hints at a common developmental origin from embryonic pallium^45^ (Supplementary Fig. 15). Furthermore, AST5 possesses a unique transcriptome, which appears to be intermediate between a progenitor (AST4) and a mature astrocyte (AST1) (Supplementary Tables 3, 4, and 6–9), consistent with the proposed concepts of cortical astrocyte formation from local proliferation of common progenitors followed by non-synchronous maturation^46–48^.

The relationship between AST2 and AST3 remains unclear at present, although a number of possibilities exist. These subtypes could be formed from a common class of progenitor, defined by early patterning events and diversified by cues from neighboring CNS cells, such as neuronal release of sonic hedgehog (Shh)^49,50^. Alternatively, they may be formed from distinct classes of progenitor, which intermix in the cortex^51^. However, in this latter scenario it is interesting to note that the morphological development of clonally-related astrocytes also appears to be strongly influenced by local environment^51^. A potential role for signals commonly associated with development (such as Shh signaling) is interesting, as evidence suggests that persistent activation of these pathways is necessary to maintain astrocyte diversity^52^. In this respect, it is not surprising that genetic ablation of neuronal *vglut1* has been reported to adversely affect postnatal development of cortical astrocytes^53^. In fact, AST2 and AST3, which are found throughout cortex, differentially express genes involved in neurotransmission. AST2 is enriched in transcripts linked to glutamatergic neurotransmission (including *Slc7a10*, which is involved in mobilization of the potent NMDA agonist d-serine^54^); in contrast, AST3 is enriched in transcripts associated with GABAergic neurotransmission. This suggests that reciprocal interactions between astrocytes and neurons may promote functional diversity at the local circuit level^39,40^. Consistent with this, a recent study on the role of astrocytes in glutamatergic synapse maturation in cortex identified astrocyte-secreted Chordin-like 1 (*Chrd1*)^42^ as an essential factor stabilizing synapses, which we find enriched in AST2. In this respect, comparative studies on the synaptogenic action of Norrin (*Ndp*) (present in ASTs 2 and 3) may prove instructive^13^. Furthermore, genetic manipulations, such as neuronal deletion of *Dab1*^9^ or *Satb2*^41^ also influence astrocyte positioning.

Recent years have seen a rapid growth in single-cell CNS studies^4,5,20,55^. These studies generally report transcriptomic differences between astrocytes originating from different brain regions or, in the case of cortex, an approximate separation in gene expression between layer 1 and the remaining layers (a detailed comparison between studies is made in Supplementary Fig. 16). In contrast, our results point towards a greater degree of intra-regional heterogeneity in the adult mouse brain. The possible causes for this discrepancy are unclear at present, but are likely related to methodological issues (such as age of animals used^4,20,55^, mRNA detection sensitivity^4,5,20,55,56^, etc.). However, we are confident that our results provide an accurate survey of astrocyte subtypes present in both the cortex and hippocampus of adult mouse brain. Sequencing several hundred cells for optimal coverage resulted in robust clustering of ASTs 1–4 (Supplementary Fig. 17). Only in the case of AST5, which is the rarest subtype we identified, do we consider that sequencing more cells could possibly reveal greater diversity. Furthermore, our results largely recapitulate earlier findings, produced using a variety of cell capture technologies and sequencing techniques, such as the differential expression of *Gfap* and *Mfge8* between upper and deep cortical layers^4,20^ (Supplementary Fig. 18). Furthermore, they show that markers previously reported for cortical (*Igfbp2* and *Sparc*)^5^ and hippocampal (*Nnat* and *Fabp7*)^5^ astrocytes are differentially expressed between ASTs 1–5 (see Supplementary Fig. 19 and online resource), suggesting that the optimized protocols used in our study actually allow for a more subtle characterization of cells.

The robustness of our data is demonstrated by consistency with a study from Bayraktar et al.^41^, which uses large-scale ISH-based mapping to study gene expression in somatosensory cortex of P14 mice (see Supplementary Fig. 19). In both studies, there was considerable overlap between astrocyte gene expression and spatial mapping. For example, *Id3+* astrocytes mapped to layer 1 (AST1), *Chrdl1+* astrocytes mapped to the middle cortical layers (AST2), and *Il33+* astrocytes were found over-represented in layers 5 and 6 (AST3). Although there were some differences, these could be explained by the age of the animals used^48^ or by differences in the methodologies employed.

Tissue dissociation has been reported to induce transcriptional changes^57^. However, the close correlation between our in silico analysis and ISH validation suggests this is of limited concern in our study, possibly due to the fact that we used a rapid one step isolation procedure run at low temperature (wherever possible). Another major concern with FACS use is tissue integrity and the loss of fine astrocyte processes (Supplementary Fig. 5a)^58^. Although some mRNAs in astrocytes are actively transported into processes for local translation^58^ this does not mean that those mRNA species will be absent from the soma. In fact, work in neurons has shown that there is no known case of a mRNA produced in the nucleus that is localized exclusively outside the cell body. Extrapolating on this, we expect sequencing the content of the soma to reflect the vast majority of the mRNAs expressed by an astrocyte^59^.

Our work provides a detailed characterization of astrocyte transcriptomic diversity and additional evidence that this can be linked to cell morphology^9,10^ and differential Ca^2+^ signaling^10^. However, there are issues that need to be resolved. For example, a proportion of astrocytes in cortical layer 6, which correlate to AST3, appear to have a distinct morphology^9^; the mechanisms underlying this unique specialization, such as local tissue architecture and signaling^52^, require further investigation. Given the complex molecular fingerprints of the subtypes we identified, such experiments will require the development of specific labeling techniques, based on intersectional genetics^60^.

In summary, we have demonstrated both inter- and intra-regional heterogeneity of astrocytes and have shown distinct cortical layering and hippocampal compartmentalization of these unique subtypes. Furthermore, we provide evidence that these subtypes possess distinct morphologies and physiologies. This work provides a highly resolved roadmap for future investigations of astrocyte heterogeneity. Freely available as an online resource, our data allow the development of testable hypotheses relating to astrocyte properties, which will ultimately allow their effects on CNS form and function to be elucidated. Such information will prove invaluable to our overall understanding of brain activity in both healthy and diseased states.

## Methods

### Animal experiments

All experiments were approved by the Ethical Research Committee of the KU Leuven and were in accordance with the European Communities Council Directive of 22 September 2010 (2010/63/EU) and with the relevant Belgian legislation (KB of 29 May 2013). C57BL/6J mice were used throughout. Mice aged to postnatal (P) day 56 were used for single cell RNA-seq. Male mice aged to P56–P60 were used for ISH experiments. Both male and female mice aged to P40–P67 were used for Ca^2+^ imaging experiments.

### Preparation of a single-cell suspension

Cortical and hippocampal astrocytes were prepared independently, at roughly equivalent circadian times. Two separate batches of astrocytes, originating from each region, were prepared, using multiple litters of mice. Cortical astrocytes were isolated from litter number 1. Hippocampal astrocytes were isolated from litter numbers 2 and 3.

Briefly, regions of interest (ROIs; cortex and hippocampus) were quickly and carefully dissected in cold Hanks’ balanced salt solution (HBSS) buffer without Ca^2+^ and Mg^2+^ (Sigma-Aldrich), under a binocular microscope. Myelinated parts were discarded, to decrease the amount of debris in the final cell suspension. Cortical cell suspensions were prepared from two littermate animals in parallel using separate tubes. Two hippocampal cell suspensions were also prepared in parallel using separate tubes; in this case, two different sets of four littermate animals were used. Tissue dissociation was run using the neural tissue dissociation kit (P) (Miltenyi Biotec)^25^. Tissue was digested at 37 °C using papain, supplemented with DNAse I. Tissue was mechanically dissociated using three rounds of trituration with 5 ml serological pipettes. The resulting suspension was then filtered through a 20 μm Nitex mesh (SEFAR) to remove any remaining clumps. Contamination by myelin and cell debris was removed by equilibrium density centrifugation. 90% Percoll PLUS (Life Sciences) in 1× HBSS with Ca^2+^ and Mg^2+^ (Sigma-Aldrich) was added to the suspension to produce a final concentration of 24% Percoll. Additional DNAse I (Worthington) was added (125 U per 1 ml), before the cell suspension was centrifuged at 300g_Av_ for 11 min at room temperature (with minimal centrifuge braking). The resulting cell pellet was resuspended in Dulbecco's phosphate-buffered saline (dPBS) (without Ca^2+^/Mg^2+^) containing 0.5% bovine serum albumin (BSA) (Sigma-Aldrich). Supernatants were centrifuged again at 300g_Av_ for 10 min at room temperature. Any pelleted cells were resuspended in 0.5% BSA/dPBS (without Ca^2+^/Mg^2+^). Cells were pooled and FACS isolated.

### FACS isolation of astrocytes

All steps were performed at 4 °C. Cells were incubated with FcR blocking reagent (Miltenyi Biotec) at a 1:9 dilution for 10 min to block non-specific binding of antibodies. This was followed by addition of antibodies specific to the cell isolation protocol. ACSA-2-PE antibody (Miltenyi Biotec, 130102365) (1:140 dilution) and anti-O1-eFluor660 (eBioscience, 50-6506-80) (1:810 dilution) were added to the cell suspension and incubated for 10 min. 0.5% BSA/dPBS (without Ca^2+^/Mg^2+^) was then added to the cell suspension as a washing step. Cells were recovered by centrifugation at 300g_Av_ for 10 min. The resulting pellet was then resuspended in 0.5% BSA/dPBS and filtered through a 20 μm Nitex mesh. The vital dye 7-AAD (eBioscience, 00-6993) (1:100 dilution) was added to exclude dead cells during FACS.

FACS was performed on a BD FACSAria III using a 100 µm nozzle. Compensations were done on single-color controls and gates were set on unstained samples. Forward scatter/side scatter gatings were used to remove clumps of cells and debris. Single ACSA-2-PE-positive/anti-O1-eFluor660-negative/7-AAD-negative astrocytes were sorted into separate wells of non-skirted 96-well PCR plates (VWR). Each plate also contained 1 well without any cell(s) (negative control), 1 well with 40 astrocytes (positive control: astrocytes), and 1 well with 40 7-AAD-negative cells (positive control: viable cells). Each well contained 4.3 µl of lysis buffer composed of 2.3 µl 0.2% Triton X-100 (Sigma-Aldrich) with 2 U μl^−1^ RNase inhibitor (Clontech), 1 µl of HPLC-purified 10 µM Oligo-dT30VN oligonucleotide (5′-AAGCAGTGGTATCAACGCAGAGTACT_30_VN-3′), and 1 µl of dNTP mix (Fermentas). Plates were kept at 4 °C during the sort, sealed immediately afterwards, vortexed, and spun down at 300g_Av_ for 30 s. Plates were stored at −80 °C until library preparation.

### Single-cell cDNA and library preparation

We used a modified Smart-seq2^21^ protocol. Briefly, samples were reverse transcribed. ERCC (External RNA Controls Consortium) control RNAs (Thermo Fisher Scientific) were added into the reverse transcription mix at a final dilution of 1:160 × 10^6^. TSO (5′-AAGCAGTGGTATCAACGCAGAGTACATrGrG + G-3′ in which the last guanosine is a locked nucleic acid) was used at 0.2 μM in the final reaction mix. Subsequent preamplification of cDNA used an ISPCR oligonucleotide (5′-AAGCAGTGGTATCAACGCAGAGT-3′) and 22 PCR cycles. cDNA was purified from the PCR mix using Agencourt Ampure XP beads (Beckman Coulter) with a modified bead:DNA ratio of 0.8 to 1. The quality of cDNA was checked by analyzing 11 single-cell libraries from each 96-well plate using a NGS Fragment High Sensitivity Analysis Kit (Advanced Analytical) and a Fragment Analyzer (Advanced Analytical). Data were analyzed using PROSize 2.0 software. The cDNA concentration was measured in every well using a Quant-iT PicoGreen dsDNA Kit (Invitrogen), using a standard protocol. A Synergy 2 plate reader controlled by Gen5 software (BioTek) was used to measure fluorescence.

Libraries were prepared using a Nextera XT DNA Library Preparation Kit (Illumina) with four sets of Nextera XT v2 index kits (sets A to D) (Illumina FC-131-2001 to FC-131-2004), using a standard protocol with minor modifications. Tagmentation was run on 0.125 ng cDNA (adjusted to a final volume of 1.25 μl) in a reaction mixture containing 2.5 μl Tagment DNA buffer and 1.25 μl of Amplicon Tagment Mix. This was followed by PCR amplification of adapter-ligated fragments, using a reaction mix consisting of 6.25 μl of Tagmentation product, 3.75 μl of Nextera PCR Master Mix, and 1.25 μl of each Index primer (N7xx and N5xx). PCR was run using a standard program consisting of 12 cycles. Libraries prepared with four different sets of index kits were then pooled and cleaned using Agencourt Ampure XP beads (Beckman Coulter). DNA was mixed with beads at a 1:0.6 ratio. Following an 8 min incubation, beads were recovered using a magnetic stand, supernatant was removed, and beads were washed twice with 80% ethanol. Beads were then dried for 10 min before DNA was eluted in 50 μl of EB buffer (Qiagen). Bead purification was repeated a second time using a 1:1 DNA:bead ratio. Size distribution of library pools was checked using a Fragment Analyzer and a NGS Fragment High Sensitivity Analysis Kit, according to standard protocols (Supplementary Fig. 5). Library pools were sequenced (75 bp paired-end reads) using a NextSeq 500 system (Illumina) and a NextSeq 500/550 High Output Kit v2 (150 cycles) (Illumina). Libraries were sequenced on average to a depth of 1 M reads per library.

### Analysis of RNA sequencing data

An initial quality check of sequenced libraries was undertaken using FASTQC 0.11.4 software. STAR 2.5.2b software was used to map sequencing reads against Release M12 (GRCm38.p5) of the mouse reference genome (Gencode), modified to take ERCC sequences (https://assets.thermofisher.com/TFS-Assets/LSG/manuals/cms_095047.txt) into account. Unique read maps were identified using STAR, after the removal of non-canonical unannotated junctions and non-canonical unannotated introns (using software specified parameters). Output alignment BAM files were then merged and sorted using Samtools version 1.4. RNA quality metrics were collected with Picard Tools version 1.140. Gene counts were generated using HTSeq version 0.6.1p1.

Clustering was done with Seurat version 1.4.0.16, run on RStudio version 1.0.136, using R version 3.4.0. Data were ln-normalized using the default Seurat method. Sixteen cells out of 2031 that passed FASTQC and additional quality measures (Supplementary Fig. 7) were discarded at this point, as they did not pass the minimal total expression threshold of 54 transcripts. High-level cell type identification was performed with a starting base of 24761 genes expressed across 2015 samples. In all, 5455 highly variable genes (ln-mean expression > 0.3 and ln-variance/mean > 0.1) were identified and used for clustering with the default Seurat pipeline. After discarding all other higher-order cell types, astrocytes were reclustered using the default Seurat method. Analysis was performed based on the expression of 13087 unique genes across 1811 astrocytes. In total, 886 highly variable genes (ln-mean expression > 0.5 and ln-variance/mean > 0.5) were identified and used for clustering. Unless stated, subsequent identification of genes overexpressed in the astrocyte subtypes, including specific marker genes, was also performed using the default Seurat pipeline. Genes were identified using a number of criteria. First, only significantly upregulated genes (*p* < 0.01) were considered. Second, genes had to be at least 1.28-fold overexpressed in the subtype of interest (when compared with all other astrocytes). This number was empirically chosen to give the best compromise between the number of marker genes identified in each subtype (allowing functional annotation) relative to background noise. Finally, markers had to be expressed in more than 25% of the cells identified as belonging to a particular subtype. These marker genes were further used for gene-enrichment and functional annotation analysis. Note that the AST2 marker *Unc13c* was found using the default PAGODA (R Scde 1.99.1 package) differential gene expression analysis pipeline^61^. It has a ln-mean expression of 0.28 and a ln-variance/mean of 0.41. Although it was not considered in our Seurat analysis, it remains the marker of choice for AST2, due to its remarkably high specificity.

To exclude a dominant role of batch effects in cluster analysis, extensive controls were performed and can be found in Supplementary Data 1 and online at https://holt-sc.glialab.org/.

Gene-enrichment and functional annotation analysis (GO, KEGG, and BioCarta) of subtype overexpressed genes were performed using DAVID^27^ version 6.8. All genes detected in astrocytes (13087) were used as the background gene set. Only pathways with *p*-values < 0.1 (EASE score; modified Fisher’s exact test) were taken into consideration. In addition, only pathways with *p* < 0.5 (Benjamini–Hochberg test; false discovery rate correction) were analyzed.

Genes identified as overexpressed in specific subtypes were also manually curated with the UniProt database (https://www.uniprot.org/)^28^ for assignment of putative gene functions.

### RNAscope fluorescence in situ hybridization

RNAscope (Advanced Cell Diagnostics, ACD) was performed as follows. Briefly, brains were quickly frozen in Optimum Cutting Temperature compound (Tissue-Tek), using isopentane chilled with liquid nitrogen. Ten-micrometer thick brain slices were prepared using a NX70 cryostat (Thermo Fisher Scientific). Sections were subsequently fixed in ice-cold 4% paraformaldehyde for 30 min. Sections were then dehydrated using a series of ethanol solutions (50–100%), before drying and incubating with Protease IV for 20 min at room temperature. Slides were washed in phosphate-buffered saline and hybridized with gene-specific probes (Supplementary Table 5) for 2 h at 40 °C in a HybEZ Oven (ACD). Non-annealed probes were removed by washing sections in 1× proprietary wash buffer. Probes were then detected via sequential hybridization of proprietary amplifiers and labeled “secondary” probes (Amp 1–Amp 4). Finally, sections were stained with 4′,6-diamidino-2-phenylindole (DAPI) and mounted using ProLong Diamond Antifade Mountant (Life Technologies).

### Imaging and data analysis

Brains were imaged using an Axio Scan Z1 microscope (Zeiss), operated by Zen 2.3 software (Zeiss). Images were acquired using standard excitation and emission filters. Images were taken in the best focal plane using a PL APO20×/NA 0.8 objective or a PL APO40×/NA 0.8 objective. Images were exported as separate TIFF files and imported into NIS-Elements software (version 5.02.00) (Nikon) for further analysis. Brain regions were defined according to the Allen Brain Atlas and were manually superimposed onto the images. The coloured areas in Figs. 4-6 and Supplementary Figs. 10 and 18 delineate the tissue regions quantified. Folded or otherwise distorted areas of tissue (non-coloured) were excluded from our analysis. Cells were defined as polygons centered on DAPI spots: each polygon had an average size ~1.3 times larger than that of the DAPI signal. Individual bright spots of fluorescence (signals higher than a background threshold) within these boundaries were counted as individual mRNA transcripts. Astrocytes were defined by expression of the pan astrocytic marker *Slc1a3* (coding for GLAST). Astrocyte subtypes were identified based on colocalization of specific marker genes. Data for each specific astrocyte subtype was collected from coronal sections generated from at least three independent animals. Robustness of the subtype distribution was checked by varying the fluorescence thresholds used to define transcripts across a range of intensities.

### Preparation of acute brain slices

Preparation followed a published protocol^62^ and was performed as follows. Animals were anesthetized using intraperitoneal administration of Nembutal (50 mg/kg). Transcardial perfusion was performed using 20 ml of ice-cold *N*-methyl-d-glucamine (NMDG)-based artificial cerebrospinal fluid (NMDG-ACSF) dissection solution, containing (in mM): NMDG 93, KCl 2.5, NaH_2_PO_4_ 1.25, NaHCO_3_ 30, MgSO_4_ 10, CaCl_2_ 0.5, HEPES 20, d-glucose 25, l-ascorbic acid 5, thiourea 2, sodium pyruvate 3, *N*-acetyl-l-cysteine 10; pH 7.4 (HCl). Osmolarity was adjusted to 305–310 mOsm/l if needed. The solution was bubbled in 95% O_2_/5% CO_2_ gas for 20 min before use and bubbling was maintained throughout the experiment. Following decapitation, the brain was swiftly removed. Coronal slices (350 µm-thick), containing the posterior cortex and/or dorsal hippocampus, were obtained using a Leica VT1200s vibratome. Slices were further hemisected and placed in a chamber containing NMDG-ACSF maintained at 33 °C. Slices were maintained under these conditions for 25 min, with the controlled reintroduction of Na^+^ achieved by gradual addition of 2 M NaCl to the chamber. Slices were then transferred to another chamber containing room temperature standard ACSF (in mM) NaCl 124, KCl 4.5, NaH_2_PO_4_ 1.25, NaHCO_3_ 26, MgCl_2_ 1, CaCl_2_ 2.5, d-Glucose 10; pH 7.4 (HCl); 95% O_2_/5% CO_2_ gas; osmolarity 305–310 mOsm/l. Slices were removed and transferred to a six-well plate for loading with dyes (see below), before being returned to the chamber. For imaging, slices were transferred to a specialized recording chamber and superfused with normal ACSF (including pharmacological reagents where appropriate) at 33 °C with a flow rate of 2 ml/min.

### Preparation and application of pharmacological reagents

Stock solutions were prepared using ddH_2_O and stored as frozen aliquots. Final working solutions were produced from thawed aliquots by dissolving at least 1000-fold in normal ACSF. TTX citrate (Tocris Biosciences) was used at a final concentration of 1 μM. (R)-(-)-PHE hydrochloride (Tocris Biosciences) was used at a final concentration of 50 μM.

### Astrocyte identification and calcium imaging

To identify astrocytes, cells were labeled with SR101. To avoid potential issues associated with high levels of SR101 loading (such as induction of seizure like activity)^63^, slices were incubated for 20 min in a six-well culture dish containing 1 µM SR101 (Sigma-Aldrich) in ACSF at 33 °C. Labeling of cells appeared homogeneous, as judged by visual inspection, with the possible exception of cortical layer 1, which is likely due to its high astrocyte density^64^. Following SR101 labeling, slices were moved to another well for loading with the calcium indicator Fluo-4AM. The dye was supplied as a 50 µg ampoule (Thermo Fisher Scientific) and was solubilized using a mixture of 7 μl dimethyl sulfoxide (DMSO), 2 μl 20% Pluronic F-127 in DMSO (Tocris Biosciences), and 1 μl 0.5% Kolliphor EL (Sigma-Aldrich) in DMSO. The ampoule was then incubated at 41 °C with constant agitation (1400 RPM) for 15 min using a thermomixer. Concentrated Fluo-4AM was then added to the ACSF in the well, giving a final concentration of 15.2 µM. Slices were loaded for 45–60 min at 35 °C. At the end of this period, excess AM dye was removed by washing in room temperature ACSF for at least 1 h. Prior to use, slices were maintained as described above.

All recordings followed the same protocol. First, a field of view containing either layer 1 of the cortex, layers 3–5 of the cortex, or region CA1 of the hippocampus was chosen. Each field of view was recorded under three sequential conditions. First, baseline activity was recorded. Next, ACSF containing TTX was bath applied for 5 min before additional imaging. Finally, ACSF containing TTX and PHE was then added with further imaging. Signals were recorded over a period of 300 s for each condition.

Live imaging of cells in acute slices was performed using a two-photon imaging system (VIVO 2-Photon platform, Intelligent Imaging Innovations GmbH), equipped with a tunable multiphoton laser (MaiTai laser, Spectra-Physics). Imaging was performed using a Zeiss Axio Examiner Z1, equipped with a W Plan-Apochromat 20×/NA 1.0 objective. To excite both SR101 and Fluo-4, the excitation wavelength was tuned to 820 nm. Signals were detected using two fast-gated GaAsP PMTs (Hamamatsu Photosensor Modules H11706). Images were 512 × 512 pixels in size and acquired at a frequency between 1.8 and 4 Hz. Acquisition was controlled using Slidebook 6 software (Intelligent Imaging Innovations GmbH). Laser power was limited to a maximum of 25 mW at the specimen. The focal plane used was usually 30–100 μm within the slice.

### Image analysis

Images were initially processed using Fiji software with standard plugins to correct for image drift and noise. ROIs, comprising the cell body plus proximal processes (when visible), were superimposed onto the images. The average fluorescence for each ROI per frame was measured and exported to MATLAB (The Mathworks), using custom-written scripts. Relative variations in intracellular Ca^2+^ were estimated as changes in Fluo-4 signal over the baseline (essentially dF/F_0_). Baseline (F_0_) was defined for each ROI to be the average fluorescence over the first 100 s of each recording. To measure Ca^2+^ transient parameters, we wrote custom scripts based mainly on the MATLAB function “findpeaks”. Ca^2+^ signals (peaks) were detected and their parameters measured, using the following thresholds: peaks were isolated when d*F*/*F*_0_ was higher than 1.15, events had a minimal width of 2 s, and there was a minimal separation between peaks of 1 s. The minimal prominence of a peak compared to its neighbors was set at 0.1 d*F*/*F*_0_. Only cells that responded to PHE were kept for analysis. This allowed us to define for each peak: the amplitude (defined as the maximum d*F*/*F*_0_ reached in the isolated peaks), peak time (the time at which the maximum amplitude of a given peak occurred), peak prominence (the amplitude of the peak over and above that of the closest neighboring peaks), and peak width (the width of the peak at the half height of the prominence). Reported values are averages per cell per treatment. We also measured the peak frequency, defined as the number of peaks per second (Hz) and the area under the curve, using MATLAB functions, for each cell under each recording condition. All the data can be found in Supplementary Data 5.

Further analysis was performed using RStudio 1.2.1335 running on R version 3.6.0. For baseline and TTX conditions, only astrocytes showing at least one Ca^2+^ transient during recordings were retained for analysis. Clustering and tSNE-based dimensionality reduction were performed on the Ca^2+^ transient parameters listed previously. Data were scaled and centered before tSNE analysis or hierarchical clustering. In the case of hierarchical clustering, the optimal number of clusters was determined using the silhouette width method, followed by visual inspection to identify the major branches of the tree. Statistical tests were performed on raw data (without scaling and centering). Normality was checked using a Shapiro–Wilk test. As the data was not normally distributed, a Kruskal–Wallis test was used to identify significant differences. A post-hoc Dunn’s test was performed to identify pairs of measurements that differed significantly, with multiple comparison *p*-values adjusted using the Benjamini–Hochberg method. For analysis purposes, data were compared between brain regions under the same experimental conditions.

### Figure preparation

Figures were prepared using Inkscape 0.92.2, GIMP 2.8.22, Adobe Photoshop CS6 13.0.1, and Adobe Illustrator CS6 16.0.3.

### Website preparation

The website was built using R shinyjs_1.0 and shiny_1.2.0 packages.

### Reporting summary

Further information on research design is available in the Nature Research Reporting Summary linked to this article.

## Supplementary information

Supplementary Information

Reporting Summary

Description of Additional Supplementary Files

Supplementary Data 1

Supplementary Data 2

Supplementary Data 3

Supplementary Data 4

Supplementary Data 5

## Data Availability

An easily searchable database for ISH and single-cell data generated in this study is available online at https://holt-sc.glialab.org/sc/. The full list of common genes, markers, DAVID analysis, sequencing count table (not normalized) and metadata are provided online as Supplementary Data, as is data from the Ca^2+^ imaging experiments. Raw sequencing data are publicly available through the GEO database (GSE114000).
