# Supplementary material for: Identification of region-specific astrocyte subtypes at single cell resolution
Source: Nat Commun. 2020 Mar 5;11:1220. doi: 10.1038/s41467-019-14198-8 (PMC7058027; doi:10.1038/s41467-019-14198-8)
Supplement: Supplementary file 1 — Supplementary Information [file 41467_2019_14198_MOESM1_ESM.pdf]

Identification of region-specific astrocyte subtypes at single cell resolution

Batiuk M.Y., Martirosyan, A. et al.

SUPPLEMENTARY INFORMATION

Supplementary Methods

Validation of the Percoll PLUS equilibrium density centrifugation step for pure astrocyte isolation: 8 weeks old CD1 wild type or Aldh1l1-eGFP transgenic mice were used to prepare cell suspensions, as described in Main Text. Following density equilibrium centrifugation (with Percoll PLUS density medium), cell pellets were resuspended in 0.5% BSA/PBS (without Ca^{2+}/Mg^{2+}) (Sigma-Aldrich). The remaining supernatants were diluted 6 times with 1x HBSS (with Ca^{2+} and Mg^{2+}) (Sigma-Aldrich) and were spun down at $300g_{Av}$ for 10 min at room temperature. Pelleted debris, with any remaining cells, was resuspended in 0.5% BSA/dPBS (without Ca^{2+}/Mg^{2+}). All samples were filtered through a 20 μm Nitex filter (SEFAR). Dead cells were excluded with the use of the viability dye 7-AAD (1:20 dilution) (eBioscience).

Flow cytometry was performed using a BD FACSCanto I (BD Biosciences), operated by FACSDiva software (version 6.1.3). Forward Scatter (FSC) and Side Scatter (SSC) gating was used to eliminate debris. Compensations were set on single color controls. Gates were based on negative control samples. Dead cells were excluded based on 7-AAD staining. Sample fractions obtained within the same preparation were adjusted to be equal in volume and sent through the flow cytometer at a constant speed to estimate the quantity of cells per fraction. At least 380,000 events were recorded per run. The experiment was repeated twice with identical results.

Validation of the FACS sorting strategy using flow cytometry: 8-10 weeks old CD1 wild type, or Aldh1l1-eGFP transgenic mice, were used. The protocols used to prepare the cell suspension and perform cell surface antibody labeling are described in the Main Text. Flow cytometry was performed as described above. Forward Scatter plots (FSC-W/FSC-A) were used to separate single cells from cell doublets and tissue clumps. At least 20,000 cells were recorded per sample. The experiment was repeated twice with identical results.

Quality control of astrocytes after FACS isolation:

Confirmation of single cell isolation: 7-9 weeks old C57BL/6J males were used in trial experiments using FACS to isolate single astrocytes. The protocols used to prepare the cell suspension and perform FACS are described in the Main Text. Following FACS, cells were plated onto glass-bottom μ-dishes (Ibidi), coated with poly-D-lysine (0.1 mg/ml, Sigma-Aldrich), and centrifuged at $300g_{Av}$ for 10 min at 4°C. This was followed by fixation using 4% PFA solution (Sigma-Aldrich) for 10 min at room temperature. DAPI (2 μg/ml final concentration) was added and cells were imaged using an IX81 microscope (Olympus) with a 20x objective and Hamamatsu ImagEM (C9100-13) camera, controlled by Xcellence acquisition software (Olympus). Dishes were imaged using both phase contrast and fluorescence microscopy. Figures were prepared using FIJI and Adobe Illustrator CS6 16.0.3.

RNA quality: Immediately following FACS, cells were pelleted at 300g_{Av} for 5 min at 4°C. RNA was then isolated using a RNeasy Plus Micro kit (Qiagen), according to the manufacturer's instructions. RIN (RNA integrity number) was measured using an Agilent RNA 6000 Pico Kit (Agilent Technologies) on a 2100 Bioanalyzer (Agilent Technologies) controlled using 2100 Expert Software, according to standard protocols.

Supplementary Note 1: Method Validation and Library Preparation

Validation of the Percoll PLUS equilibrium density centrifugation step for pure astrocyte isolation using flow cytometry. Percoll PLUS was used during equilibrium density centrifugation, as previously described[1], to robustly remove myelin (and other debris) that contaminated the cell suspension. This step was critical as we found myelin and debris to interfere with ACSA-2 staining and FACS sorting. The different buoyant densities of cells and myelin (plus other debris) meant that cells were pelleted, while the majority of myelin and debris remained in the supernatant. Care was taken to ensure that all cells were pelleted, to avoid introducing any bias into the subsequent analysis of cell types. Supplementary Figure 1 shows the results of an experiment designed to assess the distribution of viable astrocytes between the cell pellet and supernatant following centrifugation. We found that 95%+ of astrocytes were found in the pellet (irrespective of the brain region from which they were isolated). This experiment was repeated twice with identical results for both brain regions.

Validation of the FACS sorting strategy using flow cytometry. To avoid introducing bias into our analysis through isolation of a subpopulation of astrocytes, we validated our use of an ACSA-2-based isolation protocol, using a three-pronged strategy. The vast majority (>96%) of identified astrocytes, in a previously published single cell dataset, expressed *Atp1b2* - which is the epitope for the ACSA-2 antibody[1, 2] (Supplementary Figure 2a). This data was confirmed using ISH probes against *Atp1b2* and the general astrocyte marker *Slc1a3* (Supplementary Figure 2b). Flow cytometry, using a single cell suspension prepared from the Aldh1l1-eGFP mouse (which is considered to be the current gold-standard pan-astrocyte marker line)[3], showed all eGFP-positive cells isolated from cortex and hippocampus were stained by ACSA-2 (Supplementary Figure 2c).

Care was taken to avoid collection of oligodendrocytes, a fraction of which are also positive for ACSA-2 (Supplementary Figure 3a). For this reason, we excluded oligodendrocytes during FACS by staining against O1, a validated marker of mature oligodendrocytes[4], as there was no O1 labeling of eGFP-positive cells isolated from the Aldh1l1-eGFP mouse (Supplementary Figure 3b).

FACS gates were optimized to allow recovery of all astrocytes, with varying levels of ACSA-2 expression, while excluding other cells weakly labeled by the antibody (Supplementary Figure 4).

Quality control of astrocytes after FACS isolation. The quality of the single cell preparation was quality controlled in a number of ways, prior to use in library preparation.

Confirmation of single cell isolation: The quality of the cell preparation was checked to assess the number of single cells in the FACS sorted sample, relative to clumps of cells or cell debris. Adjustment of FSC/SSC gating was made to ensure that all astrocytes, irrespective of size or granularity, were collected, while avoiding extensive amounts of small debris (Supplementary Figure 4). This procedure isolated cells of high viability (85-92%), as checked using vital dye (Trypan Blue) exclusion. Cells were then attached onto plates, fixed and imaged in phase contrast, as well as with DAPI staining (Supplementary Figure 5a). For these experiments, single cells were defined as having a clearly demarcated membrane under phase contrast and a single DAPI stained nucleus. Cell debris seen under phase contrast did not show DAPI staining, whereas cell clusters contained multiple DAPI signals. Cell suspension prepared from cortex contained 77.9% single cells, 20.5% debris and only 1.6% clustered cells. Likewise, cell suspension prepared from hippocampus contained 75.2% single cells, 21.6% debris and only 3.2% clustered cells. Samples for this set of experiments were prepared in 4 separate FACS isolation procedures: 2 separate isolations per brain region.

RNA quality: The quality of RNA extracted from sorted astrocytes was checked, as an indicator of general cell health. Bioanalyzer analysis showed that RNA extracted from isolated astrocytes was of good quality, with a RIN between 7.9 and 8.6 (where 1 is fully degraded RNA and 10 is fully intact RNA) (Supplementary Figure 5b).

Adaptation of the Smart-seq2 protocol for library preparation from adult astrocytes. The standard Smart-seq2 protocol we originally used did not produce cDNA libraries of good quality[5], likely due to the low amount of RNA present in adult astrocytes[2]. To improve library quality, we systematically varied steps in the published protocol. First, we reduced the concentration of the template switching oligonucleotide (TSO) used in the final reaction mixture to 0.2 μM, thus reducing the amount of small primer concatamers present. Second, the number of cycles used in the PCR pre-amplification step was increased to 22. Finally, amplified concatamers were depleted using Agencourt Ampure XP beads in a modified ratio of beads to DNA of 0.8:1 (Supplementary Figure 6). A representative fragment analyzer electropherogram of cDNA produced from a single cell, or a pool of libraries, prepared using this modified method is shown in Supplementary Figure 5c.

Supplementary Figures.

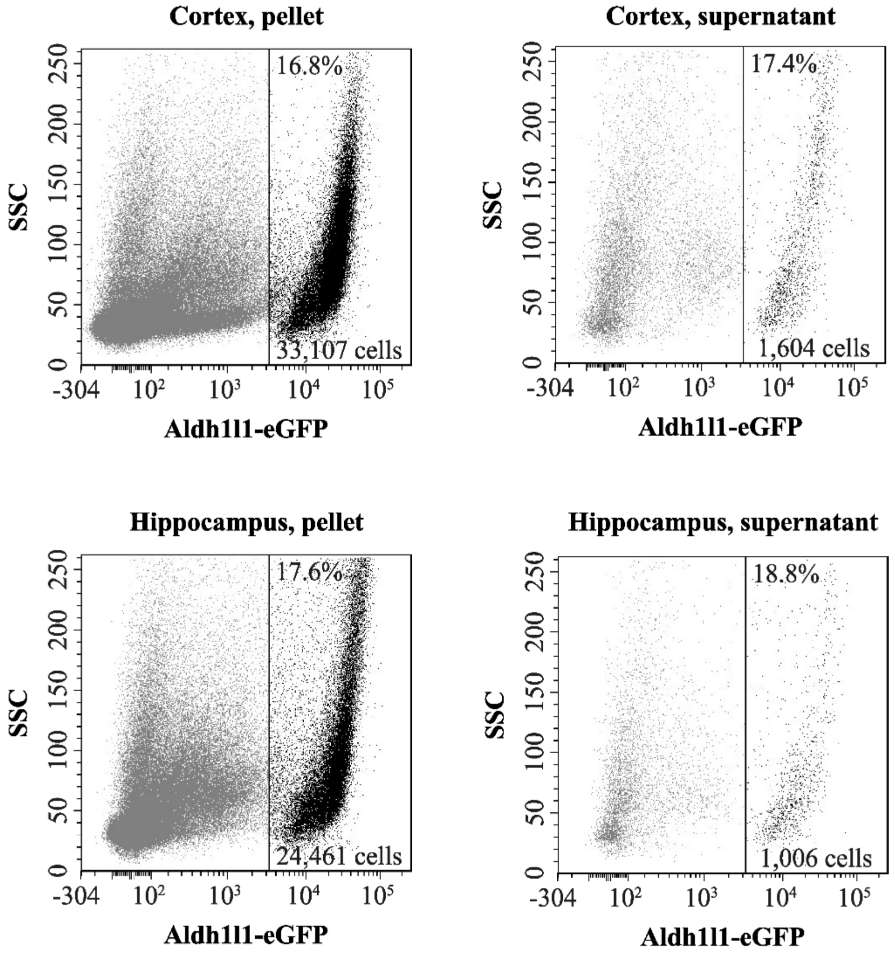

Supplementary Figure 1. Validation of the Percoll PLUS equilibrium density centrifugation step for pure astrocyte isolation. Equilibrium density centrifugation with Percoll PLUS efficiently removed myelin and cell debris, but did not affect astrocyte recovery. A single cell suspension was produced from an Aldh1l1-eGFP mouse, in which astrocytes are specifically labeled with eGFP[3]. Following centrifugation, the resulting cell pellet and supernatant (containing myelin and debris) were brought to the same volume and analyzed using flow cytometry. Aldh1l1-eGFP-positive astrocytes[3] were efficiently pelleted (>95% recovery), and were not retained in the supernatant, irrespective of whether tissue from cortex or hippocampus was used in the initial dissociation. The experiment was repeated twice with identical results. SSC: Side Scatter. The vertical line represents the gate separating Aldh1l1-eGFP-positive astrocytes from eGFP-negative cells. Numbers within the gate indicate the number of cells recorded during the run, both in absolute numbers and as a percentage of the total cell population.

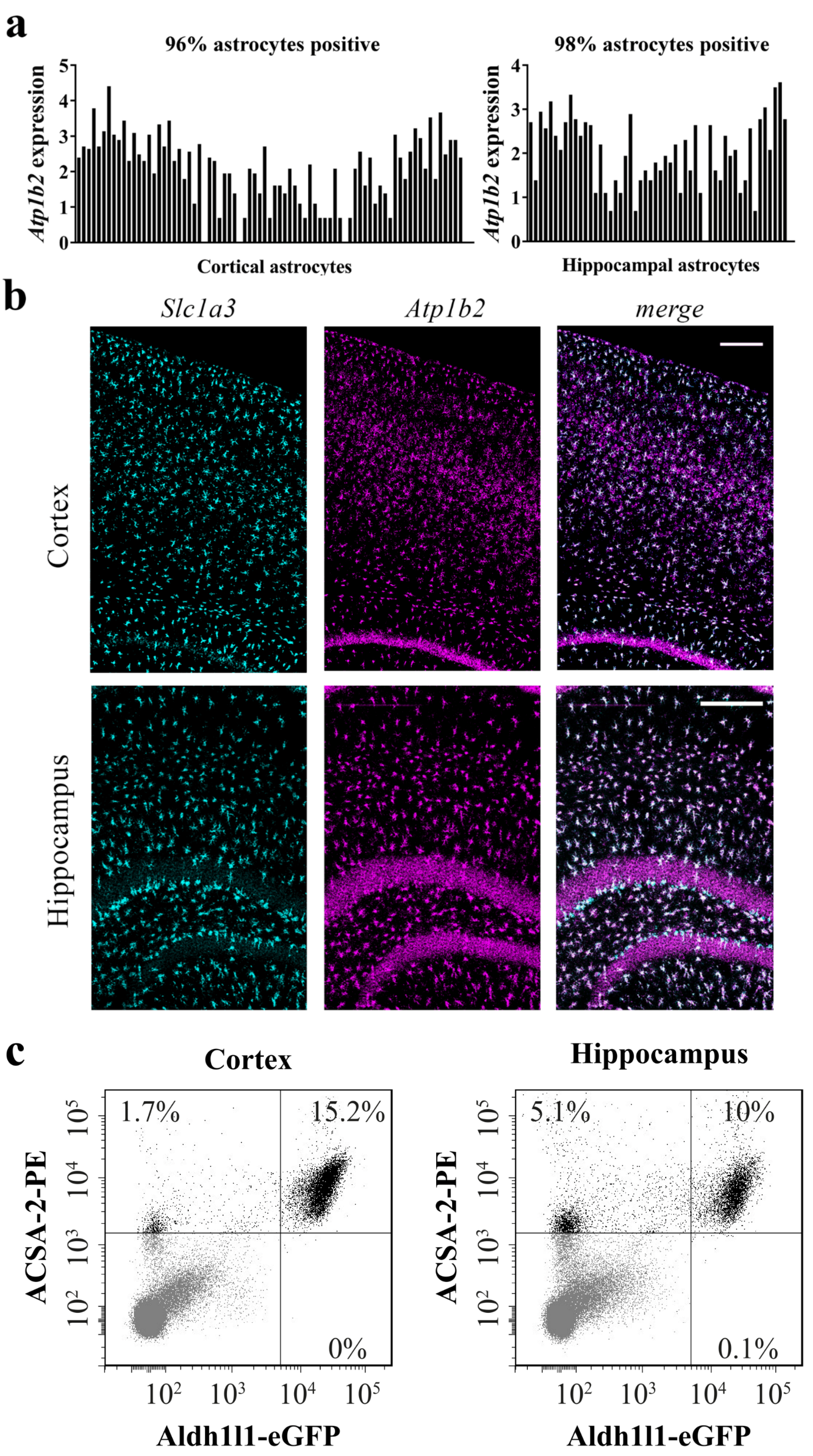

Supplementary Figure 2. ATP1B2 is found in astrocytes throughout cortex and hippocampus. (a) *Atp1b2* mRNA expression is expressed in the vast majority of astrocytes, in both cortex and hippocampus, according to the single cell transcriptome data reported by Zeisel and colleagues[2]. Data is shown in ln-scale. (b) ISH validation of *Atp1b2* expression in *Slc1a3*-

positive astrocytes. Consistent with data in (a), more than 96% of *Slc1a3* positive cells are positive for *Atp1b2*, both in hippocampus and cortex. Scale bars, 200 µm. (c) Validation of ATP1B2 protein expression in astrocytes using flow cytometry. A single cell suspension was produced from the Aldh1l1-eGFP mouse[3] (which is considered to be a pan-astrocyte marker line). The ACSA-2 antibody, which specifically targets ATP1B2[1], labels all eGFP-positive cells in the suspension. Flow cytometry experiments were repeated twice, with independent tissue preparations, and gave essentially identical results. Together, these data confirm the utility of ATP1B2 for general astrocyte isolation from both cortex and hippocampus.

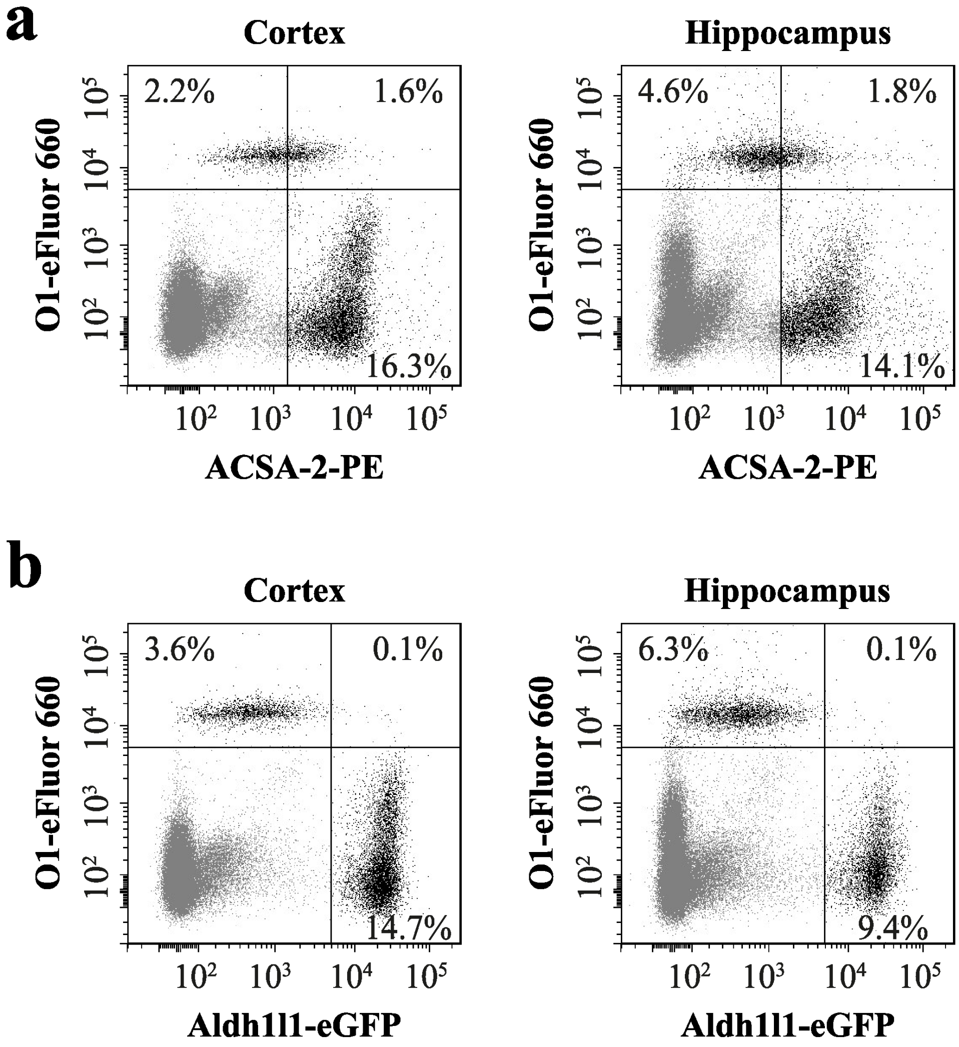

Supplementary Figure 3. Strategy for removal of oligodendrocyte contamination. (a) Cortical and hippocampal cell suspensions were stained with oligodendrocyte-specific (O1-eFluor 660) and astrocyte-specific (ACSA-2-PE) antibodies. Flow cytometry analysis revealed that a fraction of ACSA-2 labeled cells also co-stained for O1. On average, 13% of ACSA-2-positive cells were oligodendrocytes in the cortical cell suspension and 18% in the hippocampal cell suspension. O1-positive oligodendrocytes were excluded in astrocyte sorting experiments. (b) eGFP-positive cells isolated from the Aldh1l1-eGFP mouse (which is considered to be a pan-astrocyte marker line) were negative for O1-staining. Flow cytometry experiments were repeated twice, with independent tissue preparations, and gave essentially identical results.

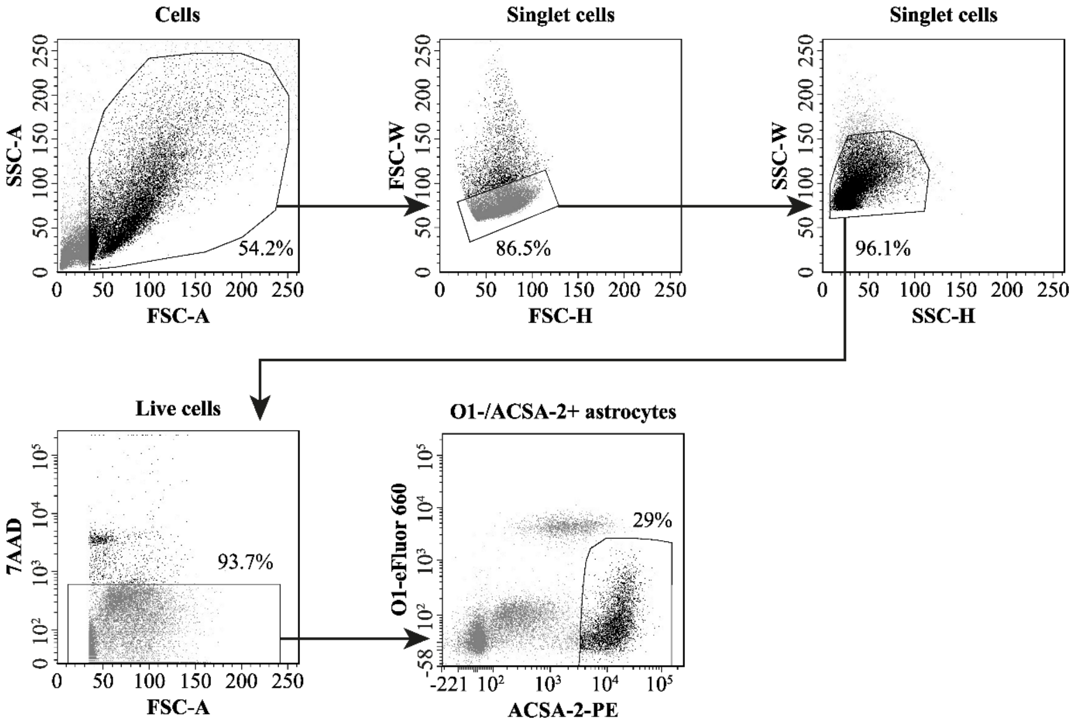

Supplementary Figure 4. FACS sorting strategy for adult astrocytes. Cells were initially selected using Forward and Side Scatter area plots (FSC-A and SSC-A). Cells were selected from inside the indicated gate. To minimize the amount of small debris collected, care was taken to adjust the lower limit on the forward scatter (measure of size) axis, although the gate was left wide enough that smaller cells were still captured. Cell doublets were excluded using forward/side scatter width vs height plots (FSC-W/FSC-H and SSC-W/SSC-H). Dead cells were excluded based on 7-AAD staining. Astrocytes were isolated as ACSA-2-PE-positive/anti-O1-eFluor 660-negative cells. Percentages represent the fraction of total cells present within the gate.

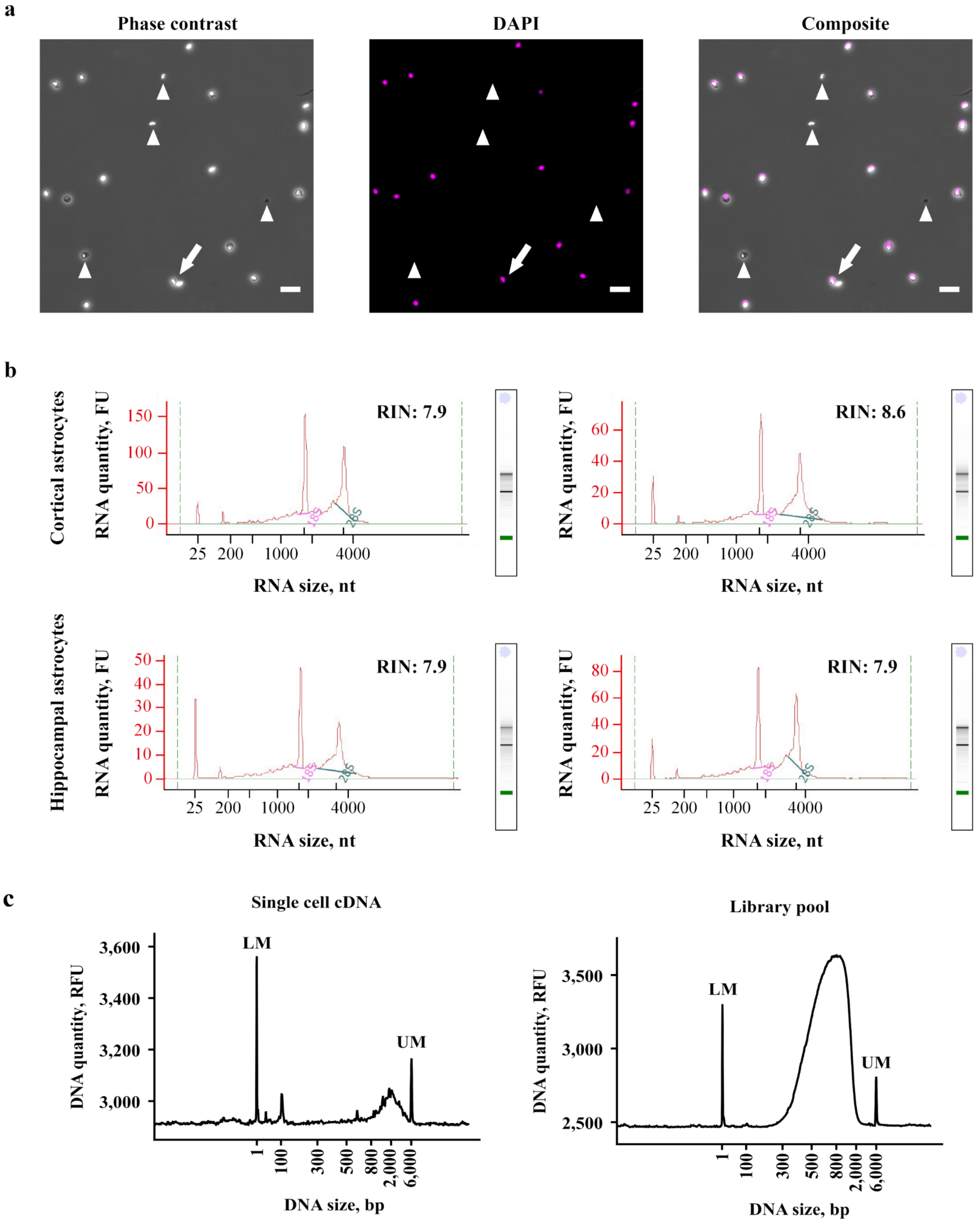

Supplementary Figure 5. Quality control of astrocytes after FACS isolation, RNA extraction and library preparation. (a) The amount of debris and cell clumps in the FACS sorted sample was checked by phase contrast and fluorescence (DAPI) microscopy. Objects without a DAPI signal were considered debris. Objects appearing as several cells clumped together in the phase contrast image and/or with multiple DAPI signals were counted as cell doublets. More than 75% of sorted objects were single cells. Arrow; cell doublet. Arrowhead; debris. Experiments were repeated twice for each brain region, with essentially identical results. Scale bar, 30 µm.

(b) The quality of RNA recovered from an astrocyte population post-sorting was checked using a Bioanalyzer. The analysis confirmed that the RNA recovered from both hippocampal and cortical astrocytes was of high quality (RNA Integrity Number (RIN) 7.9-8.6). (c) Left: Pre-amplified cDNA from a single astrocyte. Conditions for cDNA preparation were adjusted for experiments using small cells (<12 μm diameter), to simultaneously reduce the level of primer concatamers (approx. 100 bp) and improve the quantity of *bona fide* pre-amplified cDNA obtained (size between 800 – 6000 bp). Right: Pool of 384 single cell RNA sequencing libraries produced from cDNA using a Nextera XT DNA Library Preparation Kit. LM, lower marker. UM, upper marker. (R)FU; (relative) fluorescence units.

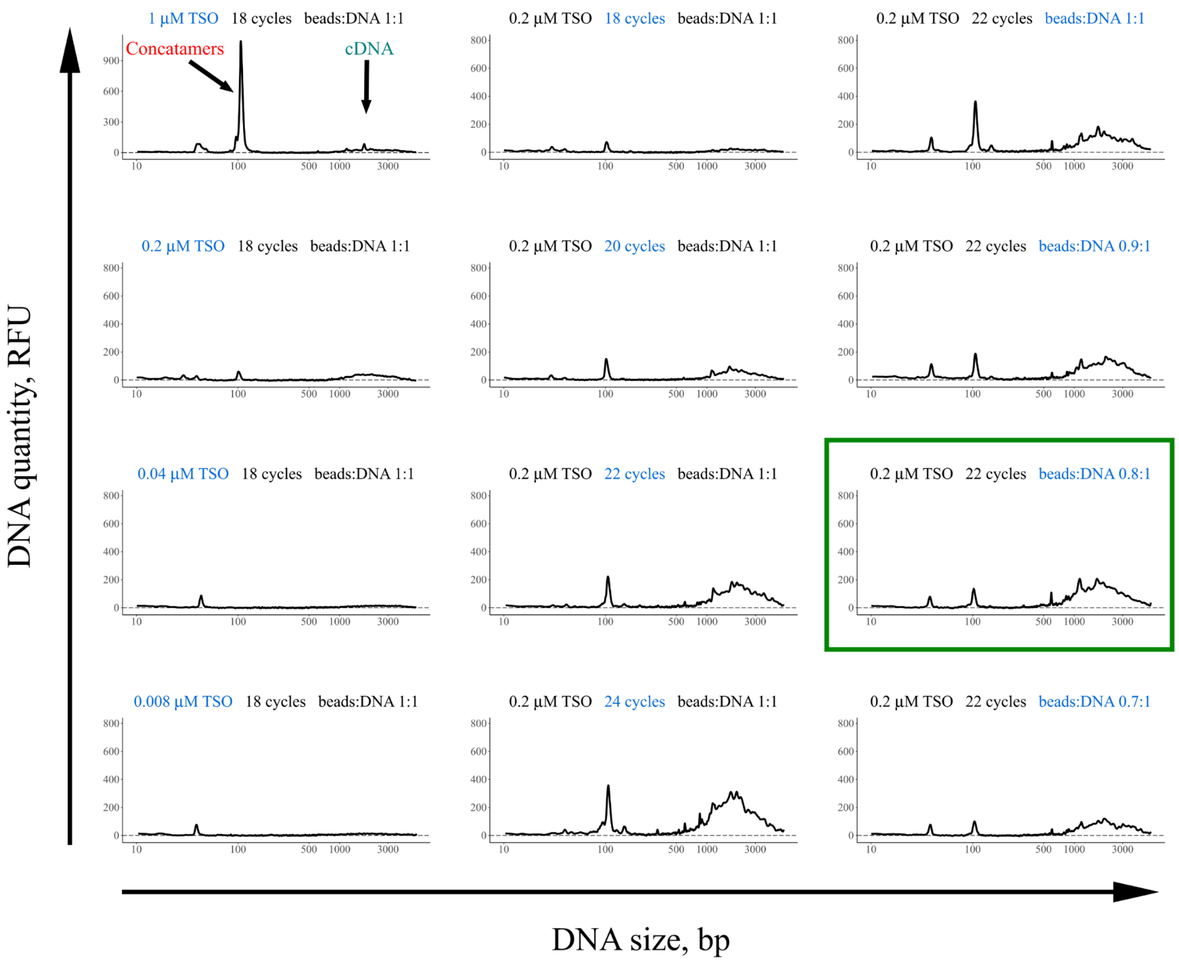

Supplementary Figure 6. Optimization of the original Smart-seq2 protocol for use with FACS sorted astrocytes. The standard Smart-seq2 protocol (top left graph) did not produce good quality single cell cDNA, as assessed using a Fragment analyzer, largely due to the low RNA content of adult astrocytes. Systematic changes to the protocol were made to reduce the amount of primer concatamers formed during the protocol and to increase the amount of cDNA produced. Three major alterations were made: optimization of the TSO concentration used to reduce concatamers (left hand column), use of a greater number of PCR cycles to increase the yield of cDNA (middle column) and optimization of the Ampure XP beads:DNA ratio to further reduce primer concatamers (right hand column). The optimal conditions identified are delineated by the green box. Upper and lower size markers are not shown. RFU; relative fluorescence units.

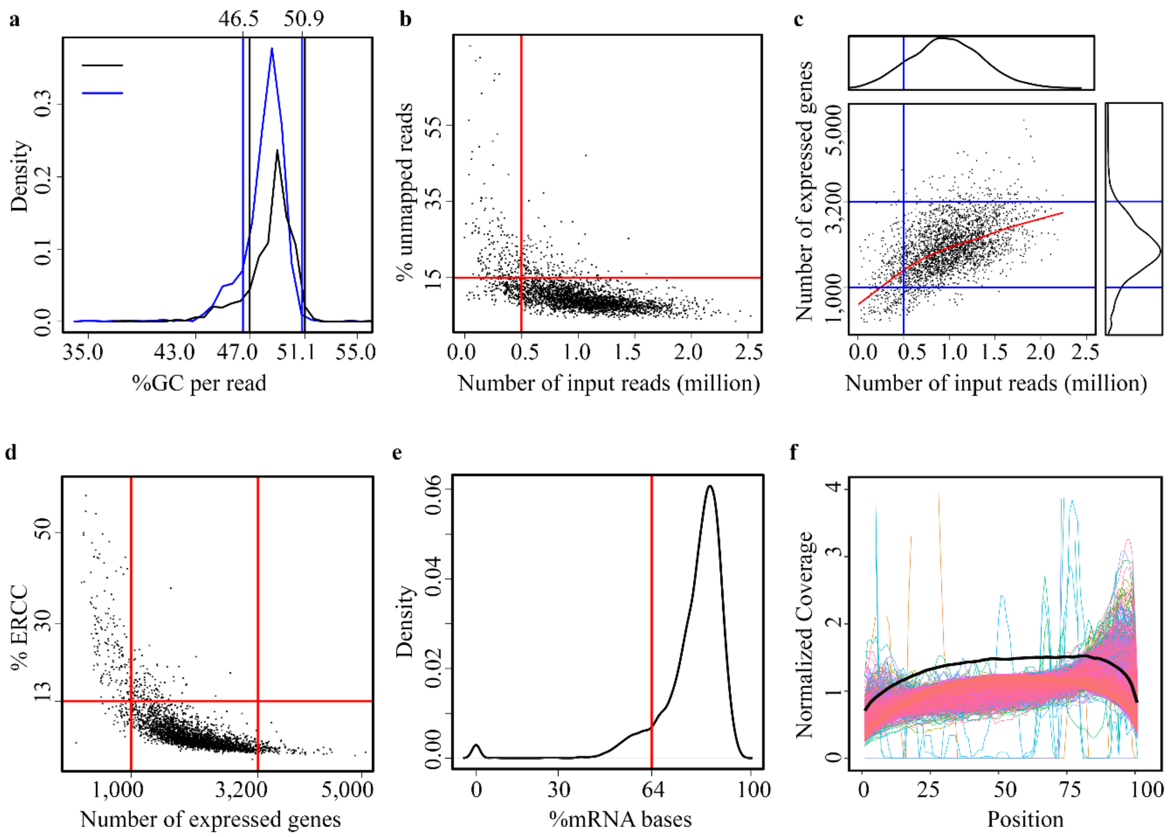

Supplementary Figure 7. Computational removal of low-quality single cell libraries. Low-quality libraries were removed based on: (a) the proportion of GC reads per transcript (falling outside the mean range of 46.5% - 50.9% or the median range of 47.0% - 51.1%); (b) the amount of input reads per library (<0.5 million) or fraction of unmapped reads per library (>15%); (c) the number of genes expressed per cell (<1000 or >3200); (d) the proportion of ERCC reads relative to total (>13%); (e) the fraction of mRNA reads per cell (<64%); (f) 5' or 3' mapping bias (0, beginning of 5' UTR; 100, end of 3' UTR). Black, blue and red lines in (a-f) show the cut-offs separating high- and low-quality libraries. The red line in (c) represents the best fit of the data. 2,015 high quality cDNA libraries passed quality control and were retained for further analysis.

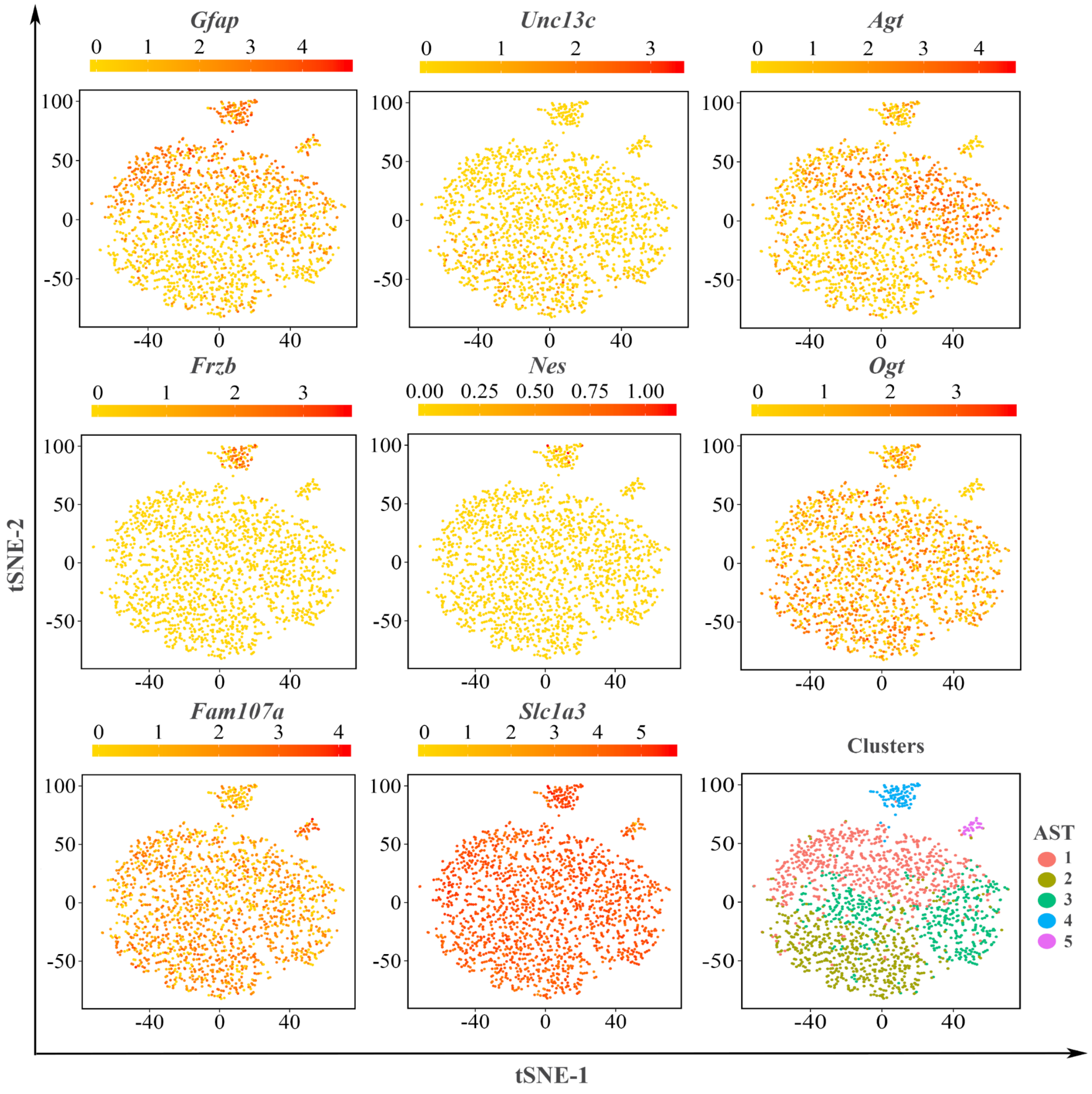

Supplementary Figure 8. Expression of subtype specific marker genes across astrocytes. Seurat-based clustering of astrocyte data identified 5 distinct astrocyte subtypes (AST), which were identified by differential expression of marker genes. Data are presented in tSNE plots.

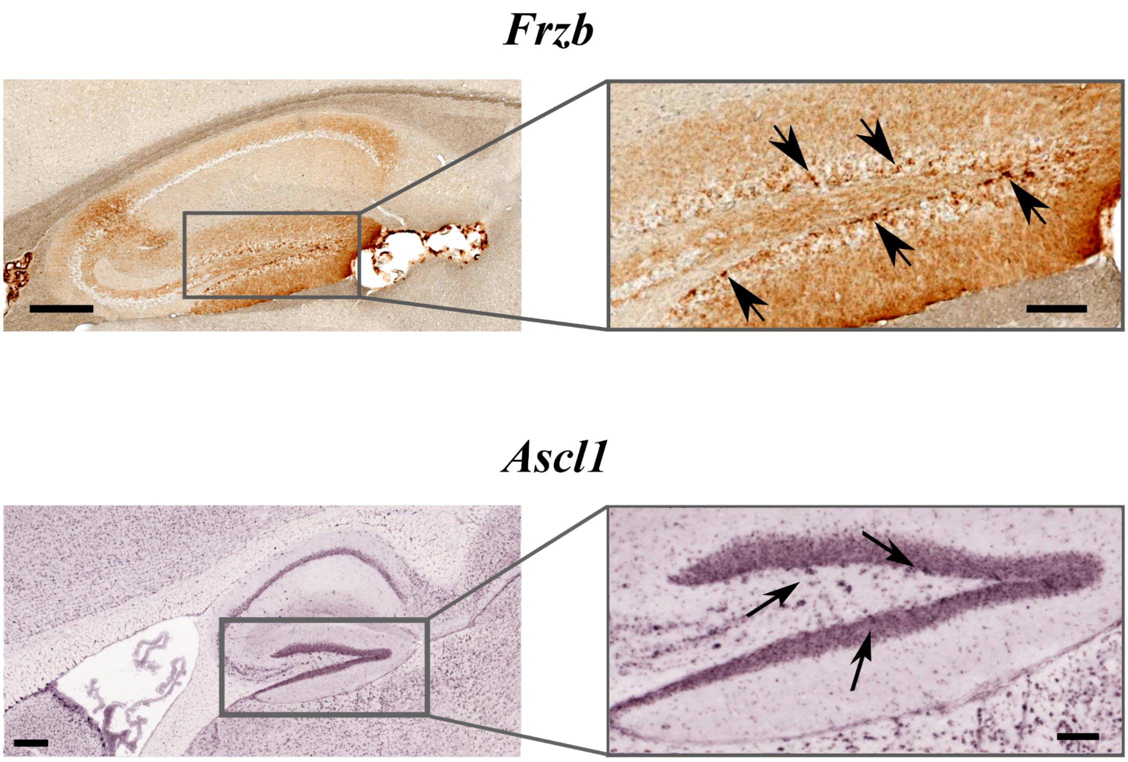

Supplementary Figure 9. AST4 maps to the hippocampus. *Frzb* and *Ascl1* were identified as markers of AST4. The GENSAT database[6, 7] is a collection of BAC transgenic animals, in which gene expression patterns are revealed by eGFP expression. Based on this data, expression of *Frzb* maps to several regions of the hippocampus, with the highest expression in cells of the subgranular zone of the dentate gyrus (top). *In situ* hybridization data from the Allen Mouse Brain Atlas[8] shows *Ascl1* is also expressed in the subgranular zone of the dentate gyrus (bottom). Based on these expression data, and the fact that *Ascl1* is known to be expressed in adult neural stem cells and amplifying progenitors[9, 10], it is likely that AST4 represents a neurogenic cell population. Arrows indicate cells expressing the genes of interest. Scale bars, left, 300 µm; right, 100 µm.

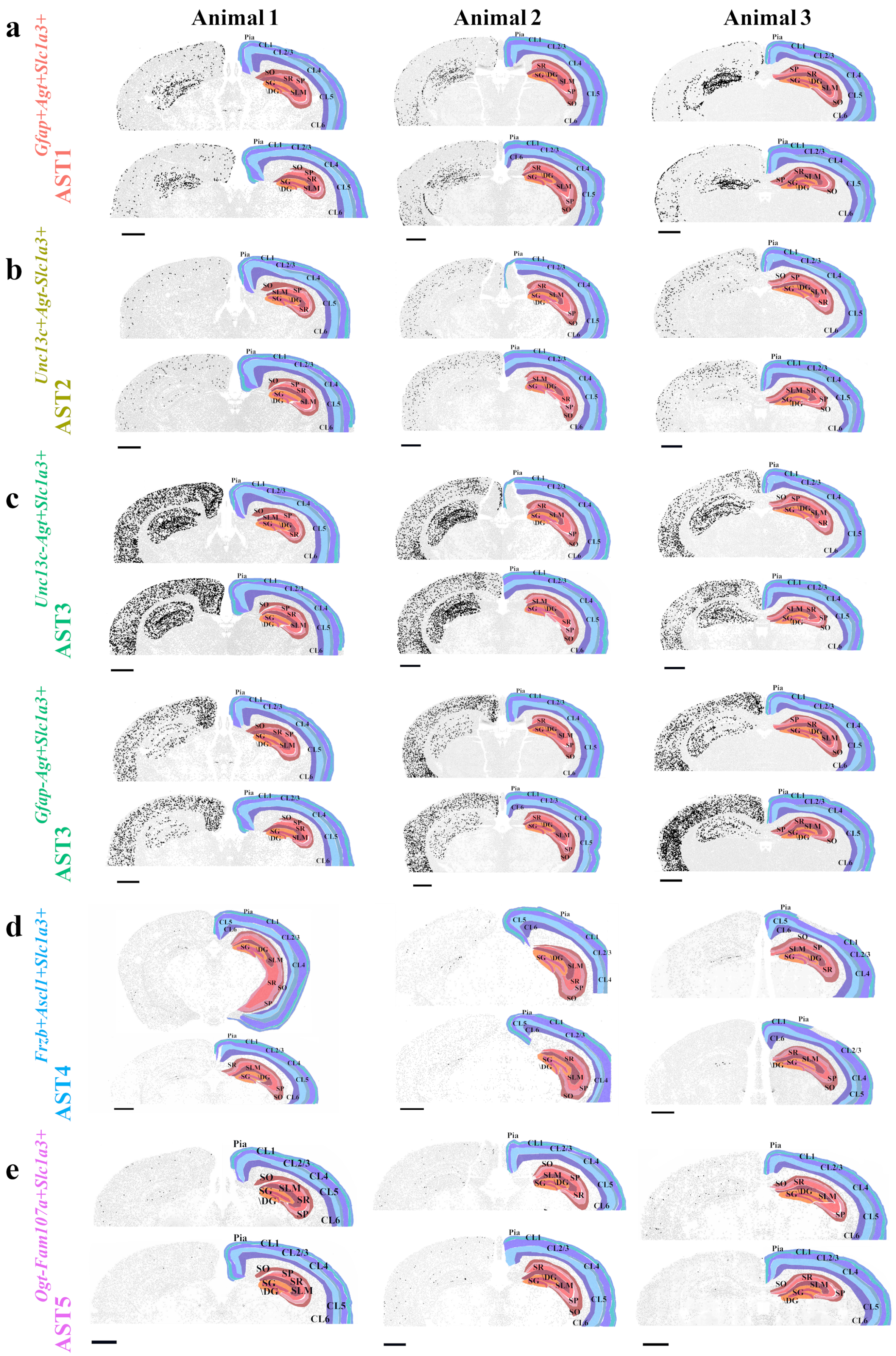
Animal 1
Animal 2
Animal 3
a
Gfap+Agt+Slc1a3+
AST1
b
Unc13c+Agt-Slc1a3+
AST2
c
Unc13c-Agt+Slc1a3+
AST3
Gfap-Agt+Slc1a3+
AST3
d
Frzb+Ascl1+Slc1a3+
AST4
e
Ogt-Fam107a+Slc1a3+
AST5
Pia
CL1
CL2/3
CL4
CL5
CL6
SO
SP
SR
SLM
SG
DG

Supplementary Figure 10. Spatial localization of astrocyte subtypes determined by *in situ* hybridization. Experiments were performed on a minimum of 3 sections, obtained from 3 individual animals, aged between post-natal days (P) 56-60. Black dots show the location of (a) AST1$^{Gfap+Agt+Slc1a3+}$, (b) AST2$^{Unc13c+Agt-Slc1a3+}$, (c) AST3$^{Unc13c-Agt+Slc1a3+}$ (top) and AST3$^{Gfap-Agt+Slc1a3+}$ (bottom), (d) AST4$^{Frzb+Ascl1+Slc1a3+}$ and (e) AST5$^{Ogt-Fam107a+Slc1a3+}$. Sections were manually segmented, based on coordinates published in the Allen Brain Atlas (Mouse Reference Atlas, Coronal). Color coding is according to Figure 2 (main text). Scale bars, 1000 µm. SO, Stratum oriens: SP, Stratum pyramidale: SR, Stratum radiatum: SG, Subgranular zone: \DG, Dentate gyrus without SG: SLM, Stratum lacunosum-moleculare.

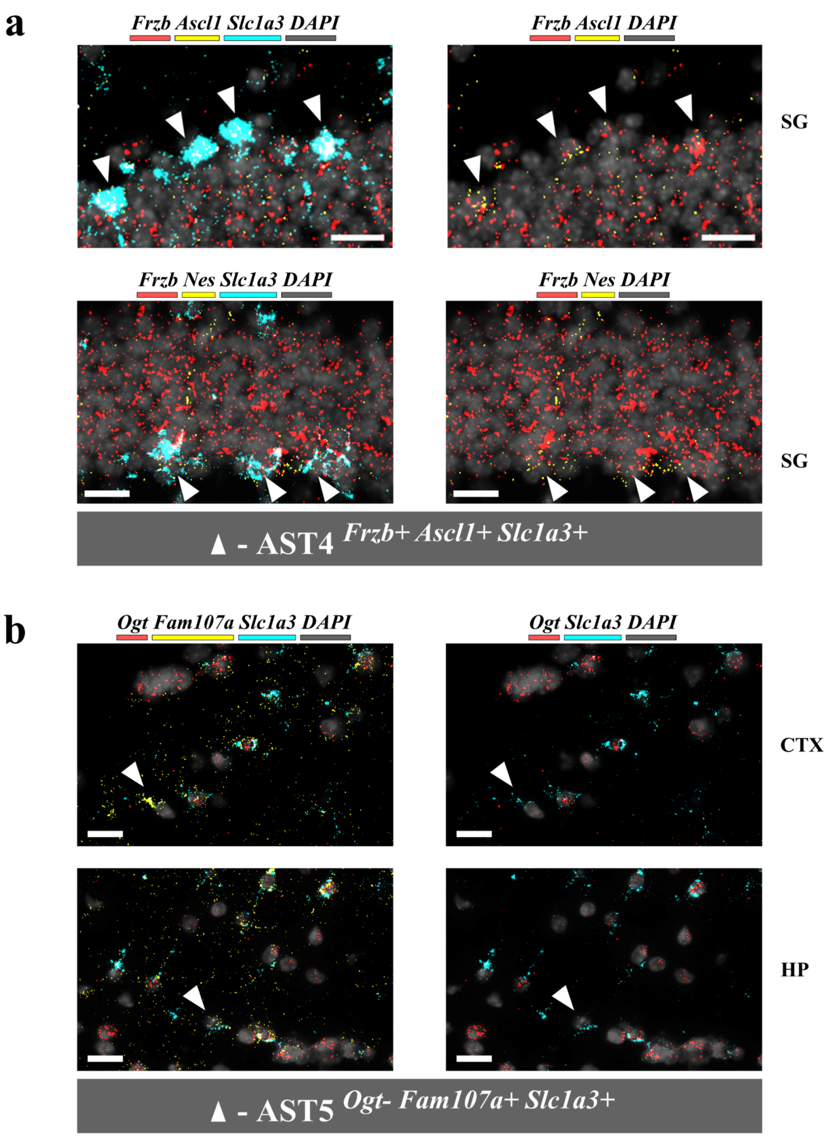

Supplementary Figure 11. Differential localization of AST4 and AST5 in adult mouse brain mapped with *in situ* hybridization. High magnification images highlighting the position of (a) AST4$^{Frzb+Ascl1+Slc1a3+}$ and (b) AST5$^{Ogt\text{-}Fam107a+Slc1a3+}$ astrocytes in brain. AST4 was detected in the subgranular zone (SGZ) of the dentate gyrus in hippocampus, which is a known site for adult neurogenesis[9, 10]. Co-staining of *Ascl1* (which is expressed in adult neural stem and amplifying progenitor cells) with *Slc1a3* (a marker of astrocytes expressed by stem cells but not generally expressed by amplifying progenitors) putatively identifies this population as adult neural stem cells[10]. This is further confirmed by co-staining with the stem cell specific marker nestin (*Nes*)[10]. AST5 is scattered through cortex (CTX) and hippocampus (HP). Scale bars, 20 µm.

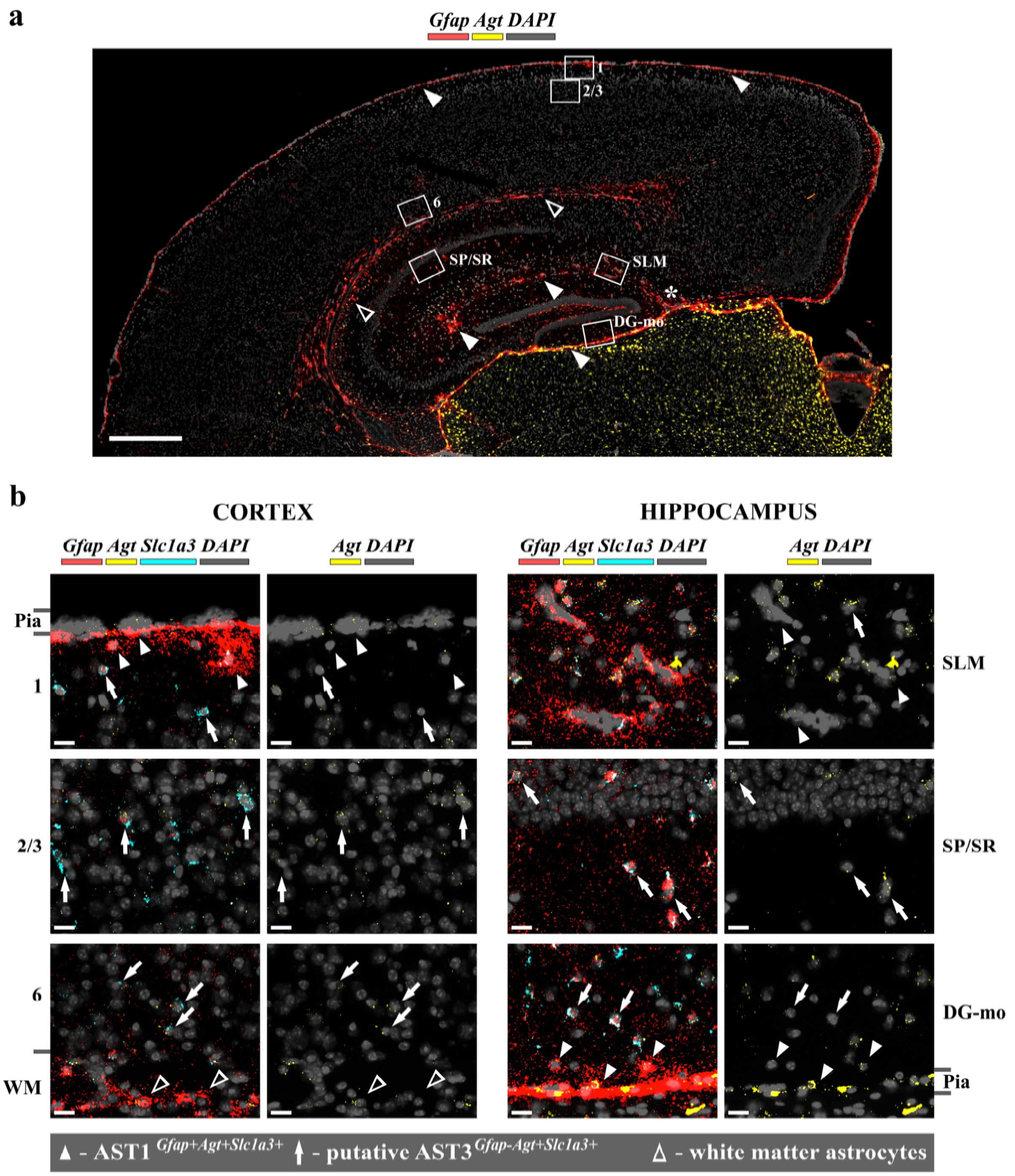

Supplementary Figure 12. Differential localization of AST1 and AST3 in adult mouse brain mapped with *in situ* hybridization. (a) Overview of a coronal section of adult mouse brain stained for AST1$^{Gfap+Agt+Slc1a3+}$. AST1 was detected in the subpial area in cortex (layer 1) and stratum lacunosum-moleculare (SLM) of the hippocampus (closed arrowheads). White matter astrocytes also highly express *Gfap* (open arrowheads), although this region was removed during tissue preparation for RNA-seq experiments. Asterisk (*) marks the hippocampal fissure. Boxes mark regions shown at higher magnification in (b). Scale bar, 500 µm. (b) High magnification images highlighting the position of AST1 astrocytes in cortex (left) and hippocampus (right) (closed arrowheads). Different cortical layers are indicated

numerically. Hippocampal regions are indicated as SLM, SP/SR (stratum pyramidale/stratum radiatum) and DG-mo (molecular layer of dentate gyrus). The position of white matter astrocytes is indicated (open arrowheads; WM). AST1 was mapped primarily to the subpial area in layer 1 of cortex, the subpial area in hippocampus and the SLM around the hippocampal fissure. Astrocytes showing expression of *Agt* and low expression of *Gfap* in cortical layers 1-6, as well as the majority of areas in the hippocampus, are likely to represent $AST3^{Gfap\text{-}Agt\text{+}Slc1a3\text{+}}$ (arrows). Scale bars, 20 μm.

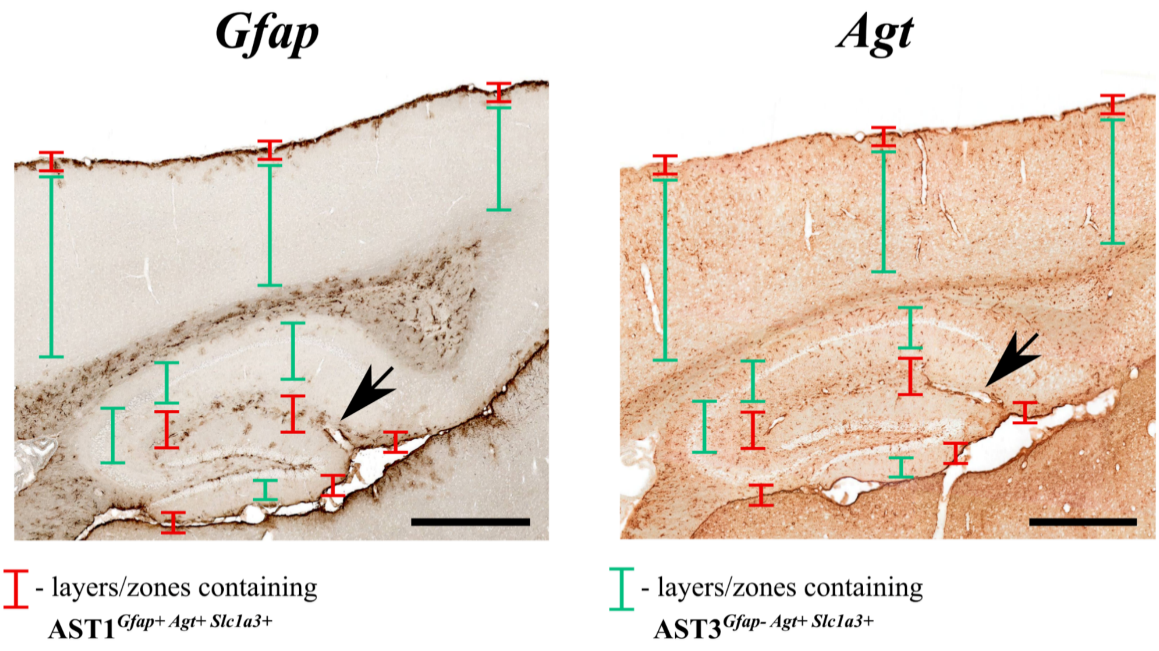

Supplementary Figure 13. Mapping the spatial distribution of AST1 and AST3 subtypes using the GENSAT database. Low resolution sagittal sections were retrieved from the GENSAT Atlas showing the eGFP expression patterns driven by the *Gfap (left)* and *Agt* (right) promoters, respectively. Based on high expression levels of *Gfap*, as well as expression of *Agt,* AST1 was mapped to layer 1 of cortex (red bars), the subpial zone in hippocampus and stratum lacunosum-moleculare around the hippocampal fissure (black arrow). AST3 (green bars) was mapped to cortical layers 1-6, stratum radiatum, stratum pyramidale, stratum oriens and dentate gyrus in the hippocampus. Note that both markers (*Gfap* and *Agt*) are also highly expressed in white matter astrocytes, which were not sequenced in this study. Scale bars, 500 µm.

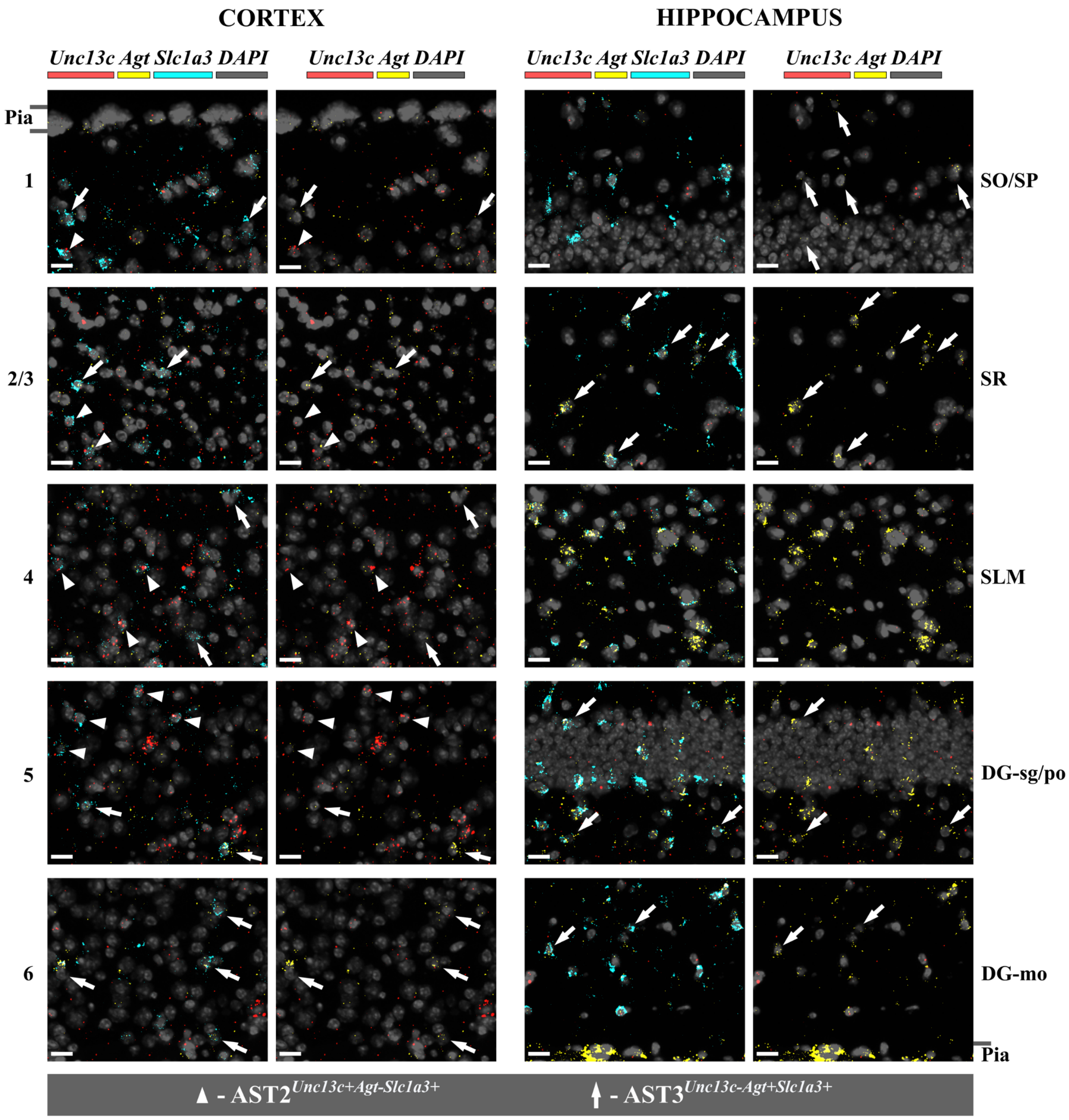

Supplementary Figure 14. Differential localization of AST2 and AST3 in adult mouse brain mapped with *in situ* hybridization. High magnification images highlighting the position of astrocytes in cortex (left) and hippocampus (right). Different cortical layers are indicated numerically. Hippocampal regions are indicated as SO (stratum oriens), SP (stratum pyramidale), stratum radiatum (SR) and stratum lacunosum-moleculare (SLM). Specific regions of the dentate gyrus, including the stratum granulosum/polymorphic (DG-sg/po) and molecular layer (DG-mo), are marked. AST2$^{Unc13c+Agt-Slc1a3+}$ was detected primarily in cortical layers 2-5 (arrowheads). AST3$^{Unc13c-Agt+Slc1a3+}$ was detected throughout layers 1-6 of cortex and SO, SP, SR, DG-sg/po and Dg-mo (arrows). Scale bars, 20 µm.

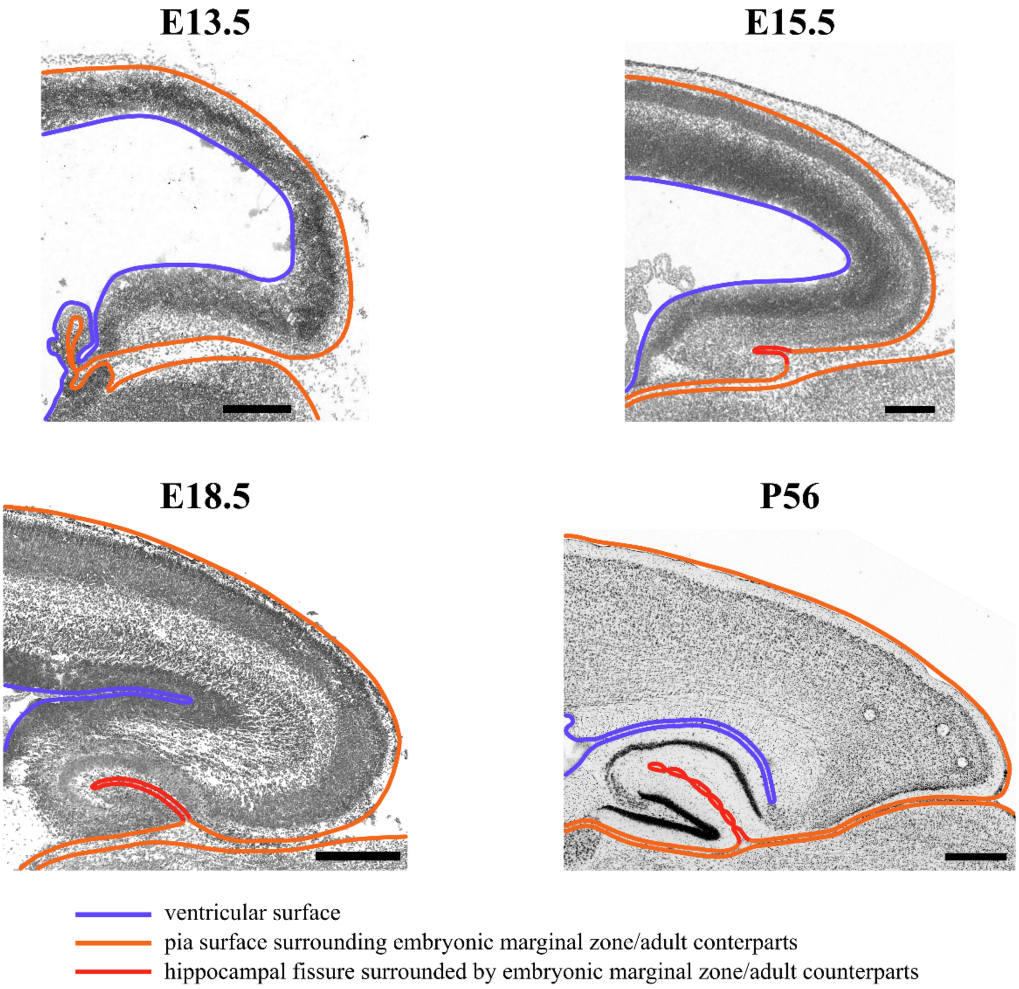

Supplementary Figure 15. Proposed scheme for shared developmental origins of cortical layer 1 and hippocampal (SLM) astrocytes. Cortex and hippocampus both develop from the embryonic pallium. At embryonic day E13.5, the hippocampal fissure is absent. The first signs of cell migration and fissure formation appear at E15.5. During later development (E18.5 and into adulthood; P56) the hippocampal fissure is surrounded by the stratum locunosum moleculare (SLM) region and separates CA1, CA2 and the dentate gyrus. Sagittal sections are displayed. Scale bars, E13.5, 280 µm; E15.5, 220 µm; E18.5, 330 µm; P56, 525 µm. Adapted from the Allen Developing Mouse Brain Atlas.

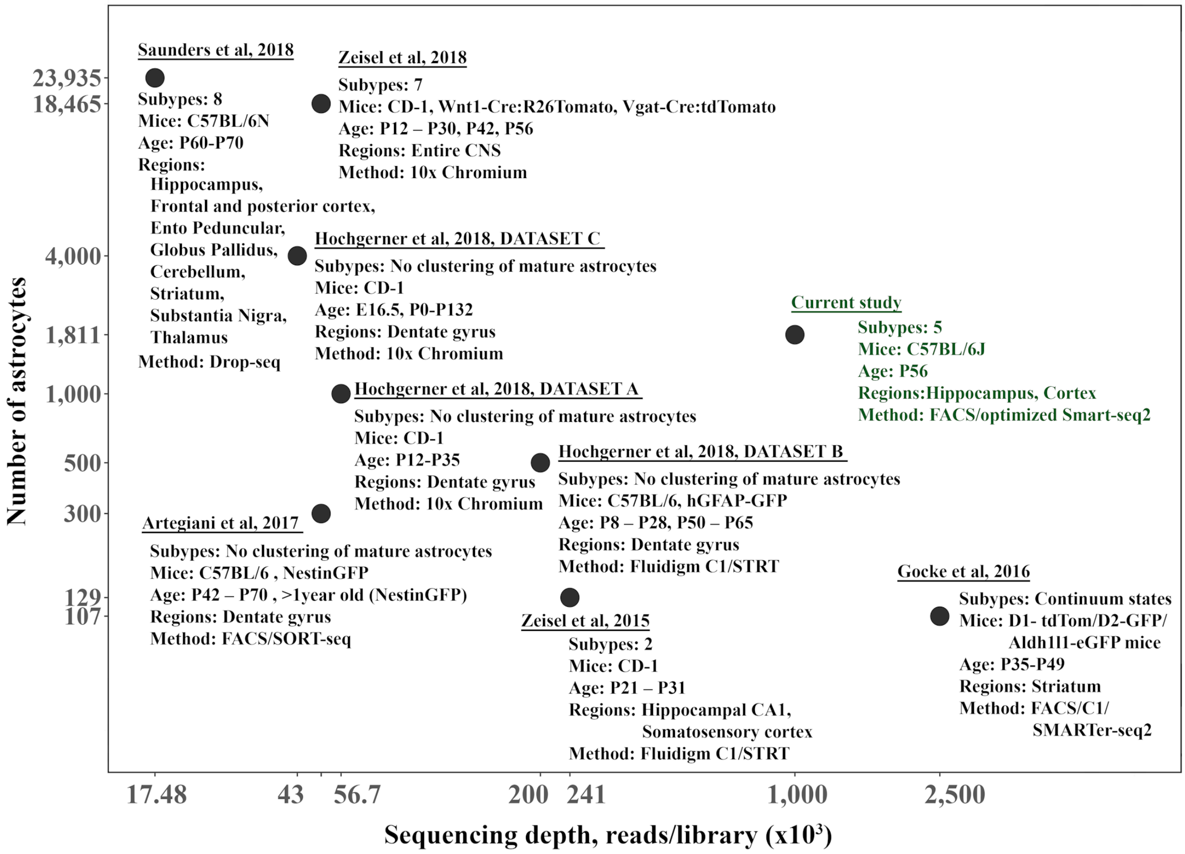

Supplementary Figure 16. An overview of astrocyte subtypes reported across major single cell sequencing studies[2, 11, 12, 13, 14, 15]. The cell isolation and library preparation methodologies used in each study are indicated, along with the brain regions sampled, mouse lines used, age of mice at time of experiment and sequencing depth. The total number of astrocytes identified in each study, as well as the number of astrocyte subtypes detected, are also reported.

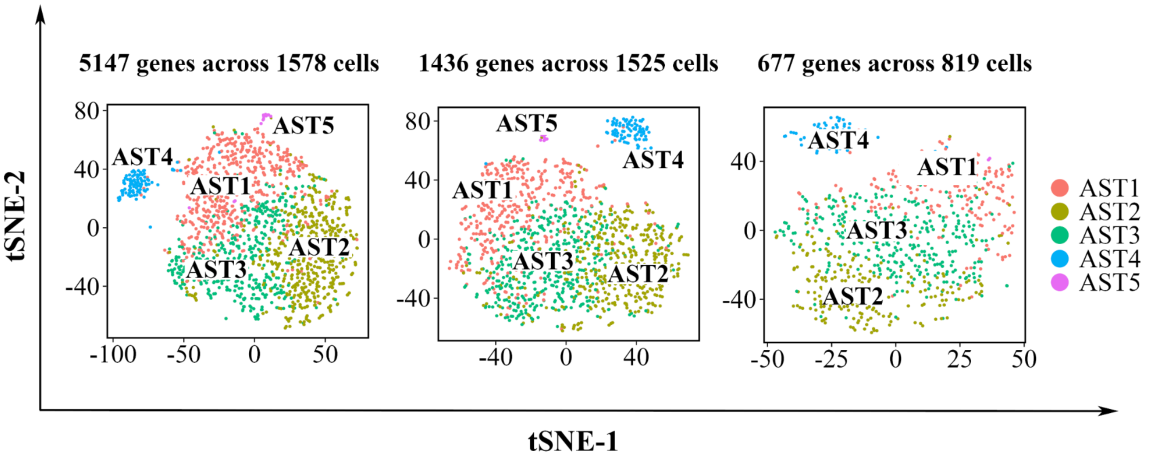

Supplementary Figure 17. Testing the robustness of Seurat clustering. A resampling approach was adopted to check clustering robustness[16, 17]. Two manipulations were made to the sampled data. First, the number of cells considered was reduced to include only those that expressed higher numbers of genes. Second, the number of genes was decreased with only those expressed in a high number of cells considered. In the main, astrocyte clusters were preserved: only the smallest initial cluster, AST5, was lost during resampling (right hand side).

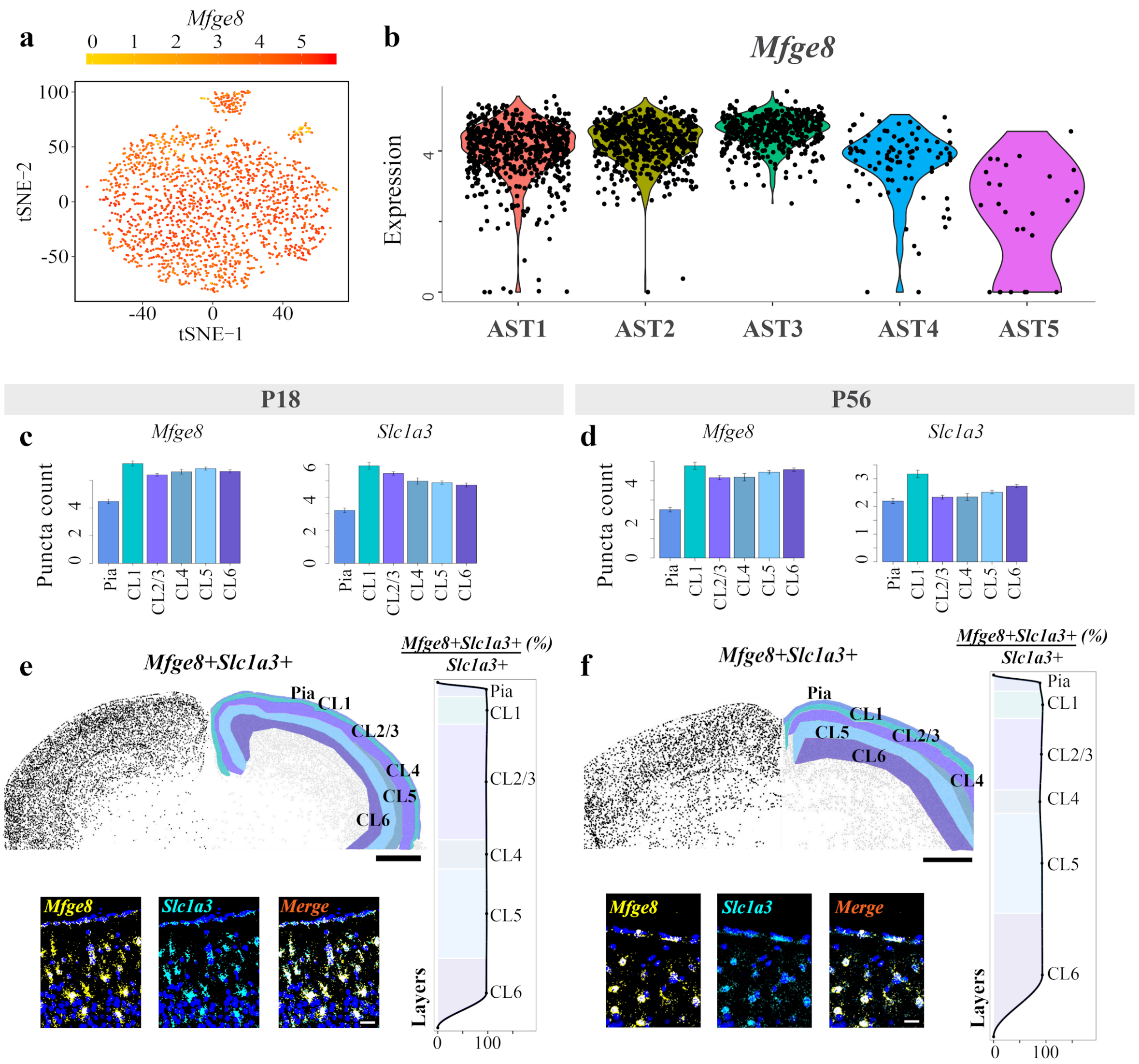

Supplementary Figure 18. Quantification of *Mfge8* expression across sampled astrocytes. (a) tSNE plot showing the expression of *Mfge8* in sequenced astrocytes (see Figure 2a for clustering). (b) Violin plots showing the ln-normalized expression of *Mfge8* across identified astrocyte subtypes. (c-f) Expression of *Mfge8* and *Slc1a3* in astrocytes at P18 (c and e) and P56 (d and f). (c, d) The average expression levels of *Mfge8* and *Slc1a3*, based on the number of fluorescent puncta imaged, were determined for *Slc1a3* positive astrocytes located at the pial surface and through the layers of the cortex. Error bars, SEM. (e, f) Sections imaged at low and high magnification for *Mfge8* and *Slc1a3*. In low magnification images, cells co-expressing the marker genes are indicated as black dots. High magnification images show the localization of markers to specific cells defined on the basis of nuclear (DAPI) staining. Plots show the distribution of astrocytes co-expressing *Mfge8* and *Slc1a3* normalized to the total number of astrocytes (all *Slc1a3*+ cells) per brain region (manually segmented according to the Allen Brain Atlas). 1 brain section was analyzed. Scale bars, low magnification 1000 µm; high magnification, 30 µm.

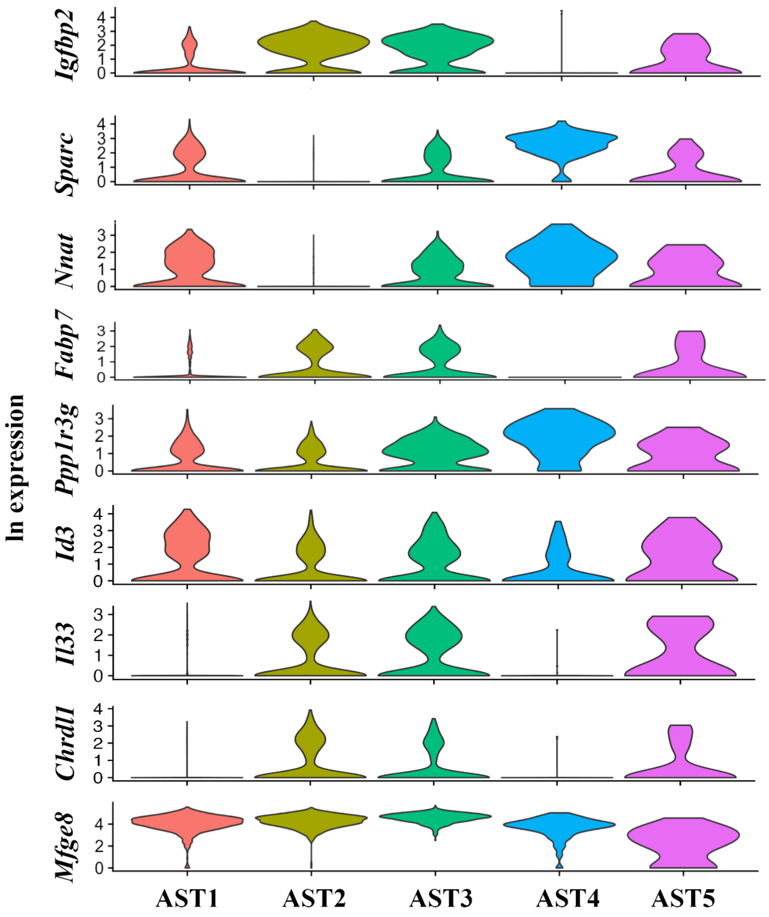

Supplementary Figure 19. Expression patterns of astrocyte subtype markers defined in other single cell studies. Expression patterns of *Mfge8*[2], *Ppplr3g*[12], *Fabp7*[11], *Nnat*[11], *Sparc*[11] and *Igfbp2*[11] are plotted across ASTs 1-5. *Chrdl1*, *Il33* and *Id3* are reported in Bayraktar et al.[18]

Supplementary Tables.

Cell type	Cortex, % of cells	Hippocampus, % of cells	Cortex and hippocampus, % of cells
Astrocytes	94.2	85.2	90.0
Neurons	0.0	1.8	0.8
Oligodendrocytes	0.2	0.7	0.4
Microglia	0.6	0.5	0.5
Endothelium	1.1	1.9	1.5
Mural cells	3.9	9.9	6.8

Supplementary Table 1. Representation of different brain cell types in FACS sorted cells. Cell types were identified by high level Seurat clustering and expression of cell type specific markers (see Figure 1 and Main Text). The vast majority of isolated cells were astrocytes. Mural cells represented the single biggest contaminating cell type.

Subtype	Number of cells	Fraction of total cells, %	Fraction of subtype cells derived from cortex, %	Fraction of subtype cells derived from hippocampus, %
AST1	661	36.5	17.6	82.4
AST2	573	31.7	94.4	5.6
AST3	460	25.4	68.0	32.0
AST4	91	5.0	2.2	97.8
AST5	26	1.4	69.2	30.8

Supplementary Table 2. Distribution of astrocytes between the identified subtypes.

% Overlapping markers	AST1	AST2	AST3	AST4	AST5
AST1	100	0	0	27.02	8.11
AST2	0	100	3.17	0	1.58
AST3	0	11.11	100	8.33	5.55
AST4	2.17	0	0.65	100	10.86
AST5	1.38	0.92	0.92	23.04	100

Supplementary Table 3. The percentage of markers genes in a given astrocyte subtype (row) shared with other subtypes (column).

Shared genes in top 10 markers	AST1	AST2	AST3	AST4	AST5
AST1	All	-	-	*Cd9*	-
AST2	-	All	*Igfbp2*	-	-
AST3	-	*Igfbp2*	All	-	-
AST4	*Cd9*	-	-	All	-
AST5	-	-	-	-	All

Supplementary Table 4. Shared genes in the list of top 10 markers for astrocyte subtypes.

Gene name	ACD product code	Fluorescence channel	mRNA accession number
Agt	426941-C2	C2	NM_007428.3
Frzb	404861	C1	NM_011356.4
Ogt	423601	C1	NM_139144.4
Gfap	313211	C1	NM_001131020.1
Slc1a3	430781-C3	C3	NM_148938.3
Unc13c	519021	C1	NM_001081153.1
Fam107a	519011-C2	C2	NM_183187.3
Ascl1	313291-C2	C2	NM_008553.4
Mfge8	408771	C1	NM_001045489.1
Nes	313161-C2	C2	NM_016701.3
Atp1b2	417131	C1	NM_013415.5

Supplementary Table 5. RNAscope probes used for *in situ* hybridization. Details of the probes used and the mRNA sequences against which they were designed are given.

Subtype	Category	Term	Gene count	*p*-value	Benjamini
**60% AST**	GOTERM_CC_DIRECT	GO:0016020~membrane	117	2.63E-15	3.52E-12
	GOTERM_CC_DIRECT	GO:0005737~cytoplasm	82	1E-03	1E-02
	GOTERM_CC_DIRECT	GO:0055114~oxidation-reduction process	16	5E-2	0.14
	GOTERM_CC_DIRECT	GO:0042632~cholesterol homeostasis	8	2E-02	0.5
	KEGG_PATHWAY	mmu00190:Oxidative phosphorylation	7	1E-02	0.16
	GOTERM_CC_DIRECT	GO:0006096~glycolytic process	5	3.75E-04	4E-02
	GOTERM_CC_DIRECT	GO:0045454~cell redox homeostasis	4	2E-02	0.5
	BIOCARTA	m_argininecPathway:Catabolic Pathways for Arginine, Histidine, Glutamate, Glutamine and Proline	3	1E-02	0.2
**AST1**	-	-	-	-	-
**AST2**	GOTERM_BP_DIRECT	GO:0008202~steroid metabolic process	6	3.71E-04	0.15
	GOTERM_BP_DIRECT	GO:0006629~lipid metabolic process	12	7.36E-04	0.19
**AST3**	GOTERM_CC_DIRECT	GO:0005902~microvillus	4	2.46E-04	0.02
	GOTERM_BP_DIRECT	GO:0032870~cellular response to hormone stimulus	5	6.45E-07	2.67E-04
**AST4**	GOTERM_BP_DIRECT	GO:0006412~translation	48	3.40E-14	7.21E-11
	GOTERM_MF_DIRECT	GO:0098641~cadherin binding involved in cell-cell adhesion	35	8.72E-10	2.71E-07
	GOTERM_BP_DIRECT	GO:0001731~formation of translation preinitiation complex	8	5.63E-05	0.02
	GOTERM_CC_DIRECT	GO:0070062~extracellular exosome	171	1.69E-28	8.27E-26
	GOTERM_MF_DIRECT	GO:0044822~poly(A) RNA binding	105	2.90E-17	1.80E-14
	GOTERM_CC_DIRECT	GO:0043209~myelin sheath	30	4.73E-11	5.78E-09
	GOTERM_CC_DIRECT	GO:0005925~focal adhesion	42	1.05E-10	1.03E-08
	GOTERM_BP_DIRECT	GO:0006457~protein folding	22	1.07E-08	1.14E-05
	GOTERM_CC_DIRECT	GO:0031012~extracellular matrix	24	1.13E-07	4.25E-06
	GOTERM_CC_DIRECT	GO:0042470~melanosome	15	1.01E-05	3.28E-04
	KEGG_PATHWAY	mmu04145:Phagosome	13	7.38E-04	0.07
	GOTERM_BP_DIRECT	GO:0008284~positive regulation of cell proliferation	24	7.59E-04	0.14
	GOTERM_CC_DIRECT	GO:0043005~neuron projection	25	1.05E-03	0.03
	GOTERM_CC_DIRECT	GO:0043197~dendritic spine	13	1.68E-03	0.04
	GOTERM_CC_DIRECT	GO:0043025~neuronal cell body	27	2.31E-03	0.04
	GOTERM_CC_DIRECT	GO:0005874~microtubule	21	2.66E-03	0.05
	GOTERM_CC_DIRECT	GO:0000785~chromatin	11	8.74E-03	0.12
**AST5**	GOTERM_CC_DIRECT	GO:0005743~mitochondrial inner membrane	34	5.30E-14	3.63E-12
	KEGG_PATHWAY	mmu00190:Oxidative phosphorylation	17	6.71E-07	2.95E-05

	KEGG_PATHWAY	mmu00010:Glycolysis / Gluconeogenesis	9	3.21E-05	9.40E-04
	GOTERM_CC_DIRECT	GO:0000421~autophagosome membrane	4	7.68E-03	0.12
	GOTERM_CC_DIRECT	GO:0005739~mitochondrion	86	2.80E-22	7.68E-20
	GOTERM_CC_DIRECT	GO:0070062~extracellular exosome	89	2.67E-18	3.66E-16
	GOTERM_CC_DIRECT	GO:0043209~myelin sheath	26	1.54E-15	1.42E-13
	GOTERM_BP_DIRECT	GO:0055114~oxidation-reduction process	31	3.77E-08	4.49E-05
	GOTERM_CC_DIRECT	GO:0005777~peroxisome	10	3.64E-04	0.01
	GOTERM_CC_DIRECT	GO:0005925~focal adhesion	16	1.61E-03	0.04
	GOTERM_CC_DIRECT	GO:0015629~actin cytoskeleton	12	1.71E-04	5.83E-03
	GOTERM_CC_DIRECT	GO:0031012~extracellular matrix	11	9.88E-04	0.02

Supplementary Table 6. Selected categories of astrocyte transcripts detected by functional enrichment analysis. Common genes (expressed in at least 60% of astrocytes) and subtype overexpressed genes were analyzed using DAVID. A cutoff ≤ 0.1 was applied to the *p*-value (EASE score, modified Fischer's exact test): an arbitrary cutoff ≤ 0.5 was used for Benjamini-Hochberg (false discovery rate; FDR) corrected *p*-values. The full list of categories identified is reported in Supplementary Data 4.

Subtype	Category	Genes	N of genes
AST4	Neural tissue development	*Lpar1, Sirt2, Lamb2, Id4, Ptprs, Dab1, Kif3a, Ascl1, Gfap, Ywhae, Ywhaq, Ywhaz, Cd9, Ndn, Fabp7, Fezf2, Ppp1ca, Cdh4, Padi2, Atxn10, Emx1, Smarcb1, H3f3b, Ywhah, Itm2c, Ephb1, Arf4, Eif5a*	28
	Mitosis and cell cycle regulation	*Sirt2, Sept2, Emp2, Sdcbp, Ran, Nudc, Mapre1, Hsp90ab1, Rhoa, Rack1, Ranbp1, Rps3, Rbbp4, Ccnd2, Calm2, Ndn, Pebp1, Ppp1ca, Btg2, Smarcb1, H2afz, Sumo1, Wdr1, Nap1l1, Pdpn, Hmgb1, Maged1, Eif5a, Ybx1, Phgdh, Cdk4, Gnai2, Nfia, Smc3, Arl3*	35
	Transcription regulators	*App, Hmgn1, Sirt2, Id4, Ascl1, Puf60, Hnrnpk, Arl2bp, Hes6, Hmgb1, Cxxc5, Maged1, Zbtb20, Ybx1, Hmgn2, Nfia, Hsp90ab1, Ywhaq, Ywhaz, Rps14, Rps3, Rbbp4, Chchd2, Fezf2, Nono, Med25, Emx1, Btg2, Smarcb1, Sri, Tcf4, Ywhah, Txn1, Maf1, Sumo1*	35
	Telomere and telomerase function and maintenance	*Hsp90ab1, Cct8, Hnrnpc, Hnrnpa2b1, Hsp90aa1, Hmgb1, Ptges3*	7
	DNA repair	*Hmgn1, Nono, H2afz, Hmgb1, Ybx1*	5
	Neurogenesis and neuron differentiation	*Lpar1, Id4, Dab1, Ascl1, Bcl2, Nudc, Ctnna1, Rhoa, Ndn, Fabp7, Btg2, Smarcb1, Tcf4, Itm2c*	14
	Genes linked to dentate gyrus and hippocampus	*Lpar1, Id4, Kif3a, Ran, Ywhae, Fezf2, Pebp1, Btg2, Ephb1*	9
	Astrocyte development	*Mapt, Dab1, Gfap, Vim, Phgdh*	5
	Oligodendrocyte development	*Lpar1, Sirt2, Ascl1, Gsn, Cd9, Kcnj10, Emx1, Wdr1, Phgdh*	9
	Cell migration	*Lpar1, Lama2, App, Arpc2, Dab1, Emp2, Ddr1, Twf1, Sdcbp, Tnc, Nudc, Ywhae, Ctnna1, Rhoa, Rack1, Cd9, Ndn, Adam15, Usp9x, Wdr1, Ephb1, Arf4, Pdpn, Ybx1*	24
	Actin cytoskeleton organization	*Lpar1, Arpc2, Emp2, Twf1, Cnn3, Dbnl, Ctnna1, Sorbs1, Gsn, Rhoa, Marcks, Capzb, Tmem47, Ywhah, Wdr1, Cdc42se2, Eif5a*	17
	Cilia organization	*Sept2, Kif3a, Gsn, Cct8, Atxn10, Dnal4, Arl3*	7
	RNA splicing	*Puf60, Hnrnpc, Nono, Hnrnpa2b1, Hnrnpf, Srsf6, Hnrnpk, Ybx1*	8
	Circadian clock	*Ubb, Ppp1ca, Nono, Hnrnpr, Maged1, Cdk4*	6
	Synaptic plasticity, memory and learning	*Mapt, Gfap, Tnc, Ccnd2, Ppp1ca, Kcnj10, Btg2, Gria1, Arf4*	9
	Synaptic function	*Lama2, Pebp1, Kcnj10, Nono, Gria1*	5
	Ubiquitination and proteasome	*Ndfip1, Rack1, Ubb, Nedd4, Usp9x, Cacybp, Sumo1*	7
	Autophagy	*Park7, Sirt2, Mapt, Ppp1ca, Pink1, Ctsl, Anxa7*	7
	Glutamatergic neurotransmission	*Gria1, Slc38a1*	2
	Ion channels	*Gria1, Kcnj10*	2
	Immediate early genes	*Fos, Jun*	2

Supplementary Table 7. Manually assigned functions for genes overexpressed in subtype AST4.

Subtype	Category	Genes	N of genes
AST5	Transcription regulators	*Ss18l2, Arl2bp, Rrn3, S100a1, Sub1, Smarca2, Taf13, Tsc22d4, Ybx1, Aip, Chchd2, Chchd3, Eef1a1, Glo1, Hsbp1, Hdgf, Id2, Med21, Nedd8, Prdx5, Ptma, Rps3, Tp53inp2, Tmbim6, Znf706, Zhx3*	26
	Neural tissue development	*Arf4, S100a1, Abi2, Cfl1, Cryab, Gpm6b, Gnb1, Id2, Pebp1, Rida, Stxbp3, Ywhae, Uqcrq*	13
	Neurite outgrowth	*Arf4, Rab10, P33monox, Rrn3, Abi2, Cfl1, Eef1a1, Gsk3b, Mgll, Plekhb1, Rhoa*	11
	Learning and memory	*Arf4, Abi2, Mgll, Dbi*	4
	Synaptic function	*Calm3, Baalc, Cript, Dbi, Gpm6b, Mgll*	6
	Formation of lamellipodia and pseudopodia, cell migration	*Carmil1, Cdc42ep4, Arpc2, Cfl1, Mapre2, Crk, Rhoa, Sdcbp, Ywhae*	9
	Lipid metabolism	*Acsbg1, Acox1, Dbi, Fabp5, Fads1, Nudt19, Pla2g16, Scp2, Mgll*	9
	Ubiquitination and proteasome	*Dda1, Skp1, Nedd8, Psmb1, Psmb5, Psmb6, Ubb*	7
	Wnt signaling	*Fermt2, Gsk3b, Snx3*	3
	Cell signaling adapter proteins	*Sdcbp, Tom1l1, Ywhae*	3
	Circadian clock	*Gsk3b, Id2*	2
	GABAergic neurotransmission	*Dbi, Gabarap*	2
	pH regulation	*Ahcyl1, Ca2*	2
	Iron metabolism	*Fth1, Ftl1*	2
	EGF signaling	*Arf4*	1
	Endocanabinoid signaling	*Mgll*	1
	Molecular chaperones	*Dnaja1, Dnaja2, Hspb8, Hspa9, Hsp90aa1*	5
	Neuron differentiation and neurogenesis	*Id2, Uqcrq, Ubb, P33monox, Gng5, Ppia, Rhoa, Sod2*	8
	Mitosis and cell cycle regulation	*Mapre2, Rps3, Tom1l1, Pebp1, Chmp2a, Cfl1, Gnl3l, Rhoa, Sh3glb1, Calm3, Sdcbp, Id2, Rida*	13
	DNA repair	*Hmgb1, Ybx1, Rps3, Hist1h2bc*	4
	Chromatin organization	*Hmgb1, Hist1h2bc*	2
	Telomere and telomerase function and maintenance	*Gnl3l, Hmgb1*	2
	Cerebral cortex development	*Rhoa, Ywhae*	2
	Hippocampus development	*Ywhae, Pebp1, Uqcrq*	3

Supplementary Table 8. Manually assigned functions for genes overexpressed in subtype AST5.

Subtype	Category	Genes	N of genes
60% AST	Oxidative phosphorylation	*Uqcrc1, Glud1, Atp5b, Atp5f1, Atp5c1, Atp5a1*	6
	Transcription factors	*Bhlhe4, Dbx2, Nr1d1, Pou3f3, Rorb, Son, Sox9, Srebf1, Tcf7l2*	9
	Homeostasis	*Slc1a3, Tmx2, P4hb, Pdia3, Prdx6, Mt1, Mt3*	7
	Glycolysis/ gluconeogenesis	*Aldoc, Pfkm, Gpi1, Gapdh, Eno1*	5
	Cholesterol metabolism	*Srebf1, Soat1, Lcat, Hmgcs1, Fdft1*	5
	Glutamate metabolism	*Glud1, Oat, Prodh, Slc1a3*	4
AST1	Synaptogenesis and neurite outgrowth	*Nrxn1, Prex2, Plekhb1*	3
	Neural tissue development	*Id3, Nnat, Plekhb1, Selenop*	4
	Iron metabolism	*B2m*	1
	Synaptic plasticity	*Agt*	1
	Transcription regulators	*Id3*	1
	Ubiquitination and proteasome degradation	*Fbxo2*	1
	Circadian clock	*Id3*	1
	Glutamatergic neurotransmission	*Arl6ip1*	1
AST2	Transcription regulators	*Xbp1, Zscan26, Tagln3, Ccnt2, Per3, Flcn, Srsf2, Wfs1, Hmgn3, Tsc22d3, Abhd14b, Chd9*	12
	Glutamatergic neurotransmission	*Slc3a2, Slc7a10, Gria2, Slc38a3, Slc25a18*	5
	Insulin signaling	*Akt2, Ogt, Rab31*	3
	Insulin-like growth factor signaling	*AI464131, Igfbp2, Akt2, Itga6, Igfbp2,*	5
	Thyroid hormone metabolism and transport	*Dio2, Slc3a2, Slco1c1*	3
	Synaptogenesis and neurite outgrowth	*Slitrk2, Slc39a12, Lrtm2, Akt2, Tnik, Sema4b, Pmp22*	7
	Synaptic plasticity and memory	*Cd47, Hrh1, Egfr, Ndrg4*	4
	Neural tissue development	*Ndrg4, Bcar3, Wls, Chrdl1, Mfsd2a, Hmgn3, Akt2*	7
	Circadian clock	*Hrh1, Ogt, Per3*	3
	Toll-like receptor signaling	*Tril, Mertk, Tlr3*	3
	Ubiquitination and proteasome degradation	*Fbxo44, Ttc3, Znrf3, Tmem129*	4
	Ion channels	*Kcnk1, Tmc7, Ttyh2*	3
	Wnt signaling	*Wls, Znrf3, Tnik*	3
	BMP signaling	*Bmp2k, Chrdl1*	2
	Notch signaling	*Bmp2k*	1
	Purinergic signaling	*Adora2b*	1
	Blood brain barrier	*Mfsd2a*	1
AST3	Transcription regulators	*Etv5, Hes5, Il33, Cyr61*	3
	Development	*Adgrg1, Fam20c, Hes5*	3

	Cell adhesion	*Adgrg1, Cyr61, Fam20c, Vcam1, Spon1*	5
	Synapse organization and neurite outgrowth	*Lrtm2, Etv5, Spon1*	3
	GABAergic neurotransmission	*Gabrg1*	1
	Carbohydrate metabolism	*Pygm, Pgm1, Ppp1r3g, Chst10*	4
	Insulin-like growth factor signaling	*Igfbp2, Cyr61*	2
	Ion channels	*Kcnj16*	1
	Synaptic function	*Trim9*	1
	Lipid metabolism	*Crot*	1
	Ubiquitination and proteasome degradation	*Trim9*	1
	Mitosis and cell cycle regulation	*Sept9, Stat3*	2
	Immediate early genes	*Fos, Jun, Dusp1*	3

Supplementary Table 9. Manually assigned functions for genes overexpressed in at least 60% of astrocytes and subtypes AST1, AST2 and AST3.

Supplementary References.

1. Batiuk MY*, et al.* An immunoaffinity-based method for isolating ultrapure adult astrocytes based on ATP1B2 targeting by the ACSA-2 antibody. *J Biol Chem* **292**, 8874-8891 (2017).

2. Zeisel A*, et al.* Brain structure. Cell types in the mouse cortex and hippocampus revealed by single-cell RNA-seq. *Science* **347**, 1138-1142 (2015).

3. Cahoy JD*, et al.* A transcriptome database for astrocytes, neurons, and oligodendrocytes: a new resource for understanding brain development and function. *J Neurosci* **28**, 264-278 (2008).

4. Yang Y, Lewis R, Miller RH. Interactions between oligodendrocyte precursors control the onset of CNS myelination. *Dev Biol* **350**, 127-138 (2011).

5. Picelli S, Faridani OR, Björklund AK, Winberg G, Sagasser S, Sandberg R. Full-length RNA-seq from single cells using Smart-seq2. *Nat Protoc* **9**, 171-181 (2014).

6. Heintz N. Gene expression nervous system atlas (GENSAT). *Nat Neurosci* **7**, 483 (2004).

7. Gong S*, et al.* A gene expression atlas of the central nervous system based on bacterial artificial chromosomes. *Nature* **425**, 917-925 (2003).

8. Lein ES*, et al.* Genome-wide atlas of gene expression in the adult mouse brain. *Nature* **445**, 168-176 (2007).

9. Pilz GA*, et al.* Live imaging of neurogenesis in the adult mouse hippocampus. *Science* **359**, 658-662 (2018).

10. Chaker Z, Codega P, Doetsch F. A mosaic world: puzzles revealed by adult neural stem cell heterogeneity. *Wiley Interdiscip Rev Dev Biol* **5**, 640-658 (2016).

11. Saunders A*, et al.* Molecular diversity and specializations among the cells of the adult mouse brain. *Cell* **174**, 1015-1030 e1016 (2018).

12. Zeisel A*, et al.* Molecular architecture of the mouse nervous system. *Cell* **174**, 999-1014 e1022 (2018).

13. Hochgerner H, Zeisel A, Lönnerberg P, Linnarsson S. Conserved properties of dentate gyrus neurogenesis across postnatal development revealed by single-cell RNA sequencing. *Nat Neurosci* **21**, 290-299 (2018).

14. Artegiani B, Lyubimova A, Muraro M, van Es JH, van Oudenaarden A, Clevers H. A single-cell RNA sequencing study reveals cellular and molecular dynamics of the hippocampal neurogenic niche. *Cell Rep* **21**, 3271-3284 (2017).

15. Gokce O*, et al.* Cellular taxonomy of the mouse striatum as revealed by single-cell RNA-seq. *Cell Rep* **16**, 1126-1137 (2016).

16. Sebastiani P, Perls TT. Detection of significant groups in hierarchical clustering by resampling. *Front Genet* **7**, 144 (2016).

17. Dudoit S, Fridlyand J. A prediction-based resampling method for estimating the number of clusters in a dataset. *Genome Biol* **3**, RESEARCH0036 (2002).

18. Bayraktar OA*, et al.* Single-cell *in situ* transcriptomic map of astrocyte cortical layer diversity. Preprint at https://doi.org/10.1101/432104 (2018).
