# Supplementary material for: Identification of region-specific astrocyte subtypes at single cell resolution
Source: Nat Commun. 2020 Mar 5;11:1220. doi: 10.1038/s41467-019-14198-8 (PMC7058027; doi:10.1038/s41467-019-14198-8)
Supplement: Supplementary file 3 — Description of Additional Supplementary Files [file 41467_2019_14198_MOESM3_ESM.docx]

**Description of Additional Supplementary Data Files**

File name: Supplementary Data 1
Description: Associated metadata describing batches during library preparations and cell identities as defined by the data analysis.

File name: Supplementary Data 2
Description: Full list of common genes (expressed in more than 60% of astrocytes) and astrocyte subtype markers identified by Seurat.

File name: Supplementary Data 3
Description: Transcript count table. Counts from 49,568 genes and 92 ERCC spike-in controls across 2,031 high quality cells that passed quality control (FASTQC and additional filtering steps shown in Supplementary Fig. 7). Columns represent cells. Rows represent genes and ERCC controls. Associated metadata is reported in Supplementary Data 1.

File name: Supplementary Data 4
Description: Full gene enrichment analysis performed using DAVID.

File name: Supplementary Data 5
Description: Ca^2+^ imaging raw data: averaged recorded peak parameters from individual cells (86 cells in baseline, 119 cells in TTX and 614 cells in PHE conditions).
